# Improving sub-pixel accuracy in ultrasound localization microscopy using supervised and self-supervised deep learning

**DOI:** 10.1088/1361-6501/ad1671

**Published:** 2024-01-09

**Authors:** Zeng Zhang, Misun Hwang, Todd J Kilbaugh, Joseph Katz

**Affiliations:** 1 Department of Mechanical Engineering, Johns Hopkins University, Baltimore, MD, United States of America; 2 Departments of Radiology, Children’s Hospital of Philadelphia, Philadelphia, PA, United States of America; 3 Department of Radiology, Perelman School of Medicine, University of Pennsylvania, Philadelphia, PA, United States of America; 4 Department of Anesthesiology and Critical Care Medicine, Children’s Hospital of Philadelphia, Philadelphia, PA, United States of America

**Keywords:** ultrasound localization microscopy, super-resolution ultrasound imaging, self-supervised learning, deep learning

## Abstract

With a spatial resolution of tens of microns, ultrasound localization microscopy (ULM) reconstructs microvascular structures and measures intravascular flows by tracking microbubbles (1–5 μm) in contrast enhanced ultrasound (CEUS) images. Since the size of CEUS bubble traces, e.g. 0.5–1 mm for ultrasound with a wavelength *λ* = 280 μm, is typically two orders of magnitude larger than the bubble diameter, accurately localizing microbubbles in noisy CEUS data is vital to the fidelity of the ULM results. In this paper, we introduce a residual learning based supervised super-resolution blind deconvolution network (SupBD-net), and a new loss function for a self-supervised blind deconvolution network (SelfBD-net), for detecting bubble centers at a spatial resolution finer than *λ*/10. Our ultimate purpose is to improve the ability to distinguish closely located microvessels and the accuracy of the velocity profile measurements in macrovessels. Using realistic synthetic data, the performance of these methods is calibrated and compared against several recently introduced deep learning and blind deconvolution techniques. For bubble detection, errors in bubble center location increase with the trace size, noise level, and bubble concentration. For all cases, SupBD-net yields the least error, keeping it below 0.1 *λ*. For unknown bubble trace morphology, where all the supervised learning methods fail, SelfBD-net can still maintain an error of less than 0.15 *λ*. SupBD-net also outperforms the other methods in separating closely located bubbles and parallel microvessels. In macrovessels, SupBD-net maintains the least errors in the vessel radius and velocity profile after introducing a procedure that corrects for terminated tracks caused by overlapping traces. Application of these methods is demonstrated by mapping the cerebral microvasculature of a neonatal pig, where neighboring microvessels separated by 0.15 *λ* can be readily distinguished by SupBD-net and SelfBD-net, but not by the other techniques. Hence, the newly proposed residual learning based methods improve the spatial resolution and accuracy of ULM in micro- and macro-vessels.

## 1. Introduction

Visualization and quantification of macro- and micro-vascular blood flows provide vital information about hemodynamic conditions which could guide timely surgical interventions for various types of diseases [1]. As a convenient bedside tool, contrast enhanced ultrasound (CEUS) imaging visualizes cardiovascular flows [2] and neonatal cerebral perfusion [3] by observing the motions of intravascular echogenic microbubbles (1–5 μm in diameter). Conventional data analysis monitors the variations in CEUS image intensity over time to estimate the perfusion [4]. Meanwhile, several postprocessing techniques have been developed to obtain quantitative blood flow information, such as ultrasound imaging velocimetry or echo particle image velocimetry [5–8], and echo particle tracking velocimetry [9–11].

Bubble tracking has also been adopted by the ultrasound localization microscopy (ULM) community [12–15], as summarized in a comprehensive review article [16]. The application of ULM to map micro-vascular structures and perfusions involves several steps. First, the microbubbles are detected and localized, which is often a challenge in noisy CEUS data. Next, they are tracked, and their trajectories are utilized for visualizing the vascular systems and measuring the flow in them. Compared to magnetic resonance imaging [17] or computed tomography [18] based techniques, which are limited to millimetric scale spatial resolutions [19], ULM can distinguish between blood vessels separated by tens of microns [15]. This technique has been used for visualizing and mapping the cerebral and renal vascular systems in rodent models [15, 20, 21]; for detecting a small deep-seated aneurysm in the human brain [19]; and for the non-invasive assessment of intracranial pressure in hydrocephalic porcine models [11].

Since the spacings between micro-vessels are typically much smaller than the size of the microbubble trace (∼500 μm) in CEUS images, the fidelity of bubble tracking is sensitive to the ability to locate the bubble center from noisy data. Given that the actual microbubble diameter is usually one order of magnitude smaller than the pixel size, the bubble trace can be modeled as a convolution between a point scatter and a spatially varying point spread function (PSF). Among the techniques developed to locate the bubble centers, some previous studies have used deconvolution procedures that estimate the local PSF [7, 22] and remove it from the bubble trace. Others have estimated the PSF as a 2D Gaussian fit to the trace of individual microbubbles and then detected bubble centers from correlations of this PSF with CEUS images [19]. In both cases, the localization accuracy is adversely affected by the spatially varying background noise, and by the presence of overlapping traces. Recent studies have shown that the deconvolution of CEUS images using end-to-end deep learning could reduce the error in bubble location by 75% [23], and could be more than 60 times faster than conventional blind deconvolution methods [24]. Similarly, superior processing speed and accuracy by deep learning methods have also been demonstrated by van Sloun *et al* [25]. The application of deep learning in ULM also involves other aspects than improving localization accuracy. For example, in a recent work [26], deep learning is used to bypass the steps of bubble locating and tracking. Instead, it performs end-to-end processing that directly converts CEUS videos to ULM results. With a slight sacrifice in spatial resolution, this method enables real-time ULM imaging with a high temporal resolution. Another work [27] utilizes a 3D convolution neural network to determine the bubble tracks from the CEUS cine-loop, which is able to reconstruct microvessels under very dense bubble concentrations. Inspired by these applications and focusing on improving the spatial resolution of ULM results, the current study introduces a super-resolution method that improves the accuracy of the measurements. Moreover, a self-supervised approach is proposed that detects bubble centers only based on raw CEUS images without reference to true bubble locations. Hence, such a method is generalizable to a broad range of CEUS imaging applications with very different PSFs.

For image super-resolution tasks, a previous study [28] has shown that the quality of results is improved by using a series of local and global residual blocks [29]. Residual learning prevents the vanishing and/or exploding gradients [30] as well as the degradation problems [31] in deep learning. Hence, it is particularly suitable for constructing very deep neural networks, leading to improved performance [29]. Consequently, residual learning has been widely applied to generate super-resolution images [32, 33]. Therefore, we adopt this approach to develop a supervised super-resolution blind deconvolution network (SupBD-net) for improving the detection of bubble centers. The primary challenges in the application of supervised learning to CEUS images are the variabilities of noise levels, backgrounds, and bubble trace morphologies, which are affected by the types of contrast agent and transducer, as well as the image settings, imaging depths, and organ structures. To overcome these issues, the training dataset should have broad characteristics, an objective achieved by generating synthetic data involving PSFs with random magnifications and orientations as well as varying background noise and bubble densities.

Finally, we introduce a self-supervised method that does not need to be trained based on references of true bubble locations by integrating ideas derived from the conventional blind deconvolution approach, namely the minimization of mean square error between the original image and the estimated bubble center convolved with the estimated PSF. This blind deconvolution approach also uses additional regularization terms based on prior knowledge that the physical size of the bubble is smaller than one pixel, i.e. it is a point scatter, hence the number of non-zero pixels around the bubble center should be minimal in the output. Mimicking this procedure, the proposed self-blind deconvolution network (SelfBD-net) uses two jointly trained networks, one for estimating the PSF, and the second, which is based on the pre-trained SupBD-net, for detecting the bubble center. The only information required for training this network pair is the raw CEUS image, hence it can be utilized for processing CEUS images with unknown PSFs.

In this paper, we aim to use super-resolution methods to improve the accuracy in detecting bubble centers out of noisy CEUS data for ULM analysis, with the ultimate goal of enhancing the ability to distinguish closely located microvessels as well as the accuracy in measuring the velocity profile and radius for macrovessels. The performances of SupBD-net and SelfBD-net are compared to those of blind deconvolution as well as several recently introduced deep learning methods, namely fully convolutional network (FCN-ULM) [34], mDensenet-ULM [35], and Deep-ULM [25]. The evaluations are based on realistic synthetic images involving a wide range of PSF morphologies, background noise levels, and bubble densities. Criteria include the error in bubble center location and the percentage of failed detections in cases of closely seated bubbles. Moreover, the performance of SelfBD-net is also tested based on synthetic images that have substantially different PSFs than the training set, in particular for cases where the supervised learning methods fail. Furthermore, the overall performance in localization and tracking is evaluated by testing the ability of these methods to distinguish between closely located parallel micro-vessels, as well as to measure the flow velocity profiles and radius of macro-vessels. The results show that the SupBD-net and SelfBD-net outperform the other methods in all tested criteria. The paper concludes by implementing and comparing the performance of each procedure using actual CEUS images obtained in the cortex of a pediatric porcine model. The methods section that comes after this introduction starts with brief descriptions of the image acquisition procedures, followed by details about each image processing method. The results and discussions are included in section 3, followed by the conclusions.

## 2. Methods

### 2.1. CEUS imaging in pediatric porcine models

The current study utilizes both synthetic and real CEUS images for demonstrating and comparing the performance of different methods. The real CEUS images are obtained from a pediatric porcine model (female, 4-week-old, 10 kg). All the animal preparation and management protocols, including anesthesia, ventilation, temperature management, cannulation, neuromonitoring, and hemodynamic monitoring, have followed previously published procedures [36] and have been approved by the Institutional Animal Care and Use Committee of the Children’s Hospital of Philadelphia. Prior to imaging, a 2 × 2 cm^2^ cranial window is drilled in the parietal region, upper right to the midline, with intact dura for mimicking the anterior fontanel of infants and obtaining clear images. A clinical scanner Siemens ACUSON Sequoia (Siemens Medical Solutions, USA) with a 9EC4 transducer (Siemens Medical Solutions, USA) is utilized to acquire images. This transducer operates at a center frequency of 5.5 MHz, corresponding to a wavelength (*λ*) of 280 μm. The default exported images have a pixel size of 60 μm, i.e. they have a resolution of *λ*/4.6. To operate at a resolution of the same order as recently published studies [25], this work further increases the spatial resolution by three times using newly proposed super resolution methods, resulting in a pixel size of 20 μm or *λ*/14. The field of view is aligned with the coronal plane containing the maximum transverse diameter of the bilateral thalami. Subsequently, the field of view is reduced to the right hemisphere to increase the image acquisition rate to 48 frames per second (fps). To minimize the relative motions between the ultrasound transducer and the animal, both the piglet’s head and the ultrasound probe are fixed to the experimental table. The undiluted microbubbles (Lumason, Bracco Diagnostics, NJ, USA) are infused at 0.6 ml min^−1^ (∼6 × 10^7^MBs min^−1^ [37]) using a veterinary syringe pump (Practivet, AZ, USA) connected to the femoral vein line. The infusion rate is selected, based on a pre-study, to establish a preferred bubble concentration of about 0.4–0.5 mm^−2^, which is low enough to facilitate the detection and tracking of individual bubbles while still maintaining a sufficient number for fully mapping the vascular structures. Image acquisition starts once the bubble reaches a steady concentration in the field of view, typically 75 s after the infusion is started, and ends at 150 s. Once CEUS images are obtained, the bubble locations are detected using the procedures described in sections 2.2, 2.4–2.6 and then tracked by an in-house developed multi-parameter global-optimized Kalman tracker [11].

### 2.2. Blind deconvolution

**Figure 1.**
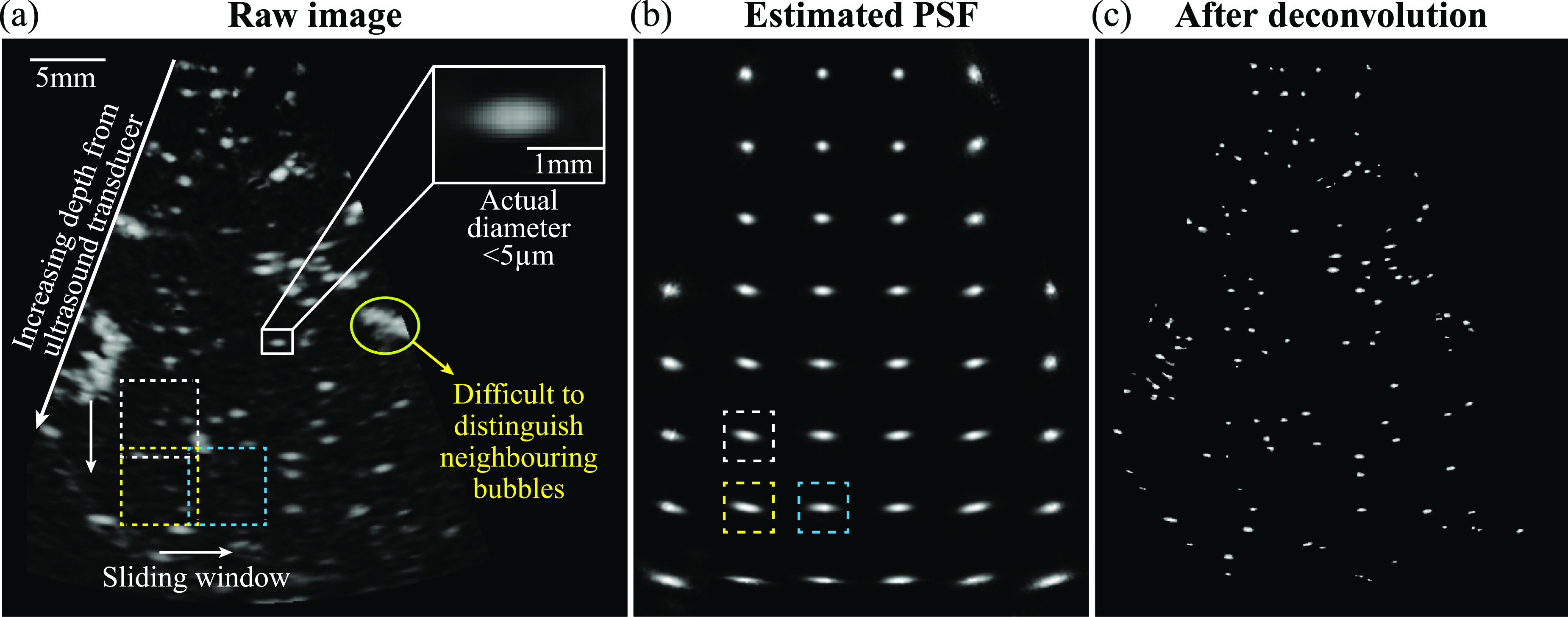
Illustration of the problems in raw CEUS images and sample results of blind deconvolution. (a) A sample raw CEUS image of a porcine’s coronal brain section. A bubble trace (white box) is magnified at the top right to show its millimetric size, which is much larger than its physical size (∼5 μm). A group of closely seated bubbles are marked in the yellow circle showing the difficulty in distinguishing them. Showing in the dashed boxes are the moving interrogation windows used for estimating the local PSF. (b) The corresponding estimated spatial distribution of PSF. Highlighted in the white, yellow, and blue dashed boxes are the PSFs corresponding to the same windows in (a). (c) A sample enhanced image after blind deconvolution.

Figure 1(a) is a sample CEUS image of the coronal brain section recorded using the Siemens scanner. As highlighted in the white box, the typical full width at half maximum (FWHM) of a bubble trace is in the order of hundreds of microns, much larger than the physical size of the contrast agent (∼5 μm). Consequently, closely located bubbles cannot be easily distinguished, as demonstrated in the yellow encircled area. Exacerbating the analysis is the tendency of the bubble trace to become increasingly elongated with increasing depth in the direction perpendicular to the scanline. Yet, obtaining precise localization for closely seated bubbles is vital for the reconstruction and flow measurement involving neighboring micro blood vessels that are separated by tens of microns. Since the bubble is an order of magnitude smaller than the pixel size of the imaging system, in prior studies, the CEUS image has been modeled as distributed point scatters blurred by spatially varying PSFs with added noise [7, 13, 38]. This model can be expressed as:

\begin{align*}\boldsymbol{y} = \boldsymbol{k} \otimes \boldsymbol{x} + \boldsymbol{n}\end{align*}

where ***y*** is the raw CEUS image, ***k*** is the 2D PSF, ***x*** is the distributed point scatters with values of one being bubble centers and zero otherwise, $ \otimes $ denotes the convolution operator, and ***n*** is the noise matrix. To determine the spatially varying ***x*** and ***k***, one can minimize the cost function ${\mathcal{L}_{\boldsymbol{BD}}}$ defined as:

\begin{align*}{\mathcal{L}_{\boldsymbol{BD}}} = \left\| {\boldsymbol{x} \otimes \boldsymbol{k} - \boldsymbol{y}} \right\|_2^2 + \gamma \left\| \boldsymbol{k} \right\|_2^2 + {\alpha _1}{\left\| \boldsymbol{x} \right\|_0} + {\alpha _2}{\left\| {\nabla \boldsymbol{x}} \right\|_0}\end{align*}

in small sub-areas of the raw image where the variations of PSF are negligible. The last three terms on the right side are regularization terms, where ${\left\| {\boldsymbol{x}} \right\|_0}$ and ${\left\| {\boldsymbol{k}} \right\|_2}$ are the *L*_0_ and *L*_2_ norms, and *γ, α*_1_, and *α*_2_ are assigned weights, whose values are selected empirically based on previous studies [39]. The use of two *L*_0_ norms is designed to ensure that the resulted ***x*** contains a minimal number of non-zero values and gradients, i.e. it is reduced to distributed point scatters. In this work, to obtain the spatial distribution of the PSF (figure 1(b)), the ${\mathcal{L}_{\boldsymbol{BD}}}$ is solved within interrogation windows of 80 × 80 pixels with a 10% overlap between neighbors sliding across the raw image (dashed squares in figure 1(a)). The window size is selected such that the PSF variations within each window can be ignored. Deconvolving the raw image with these PSFs at the corresponding locations provides the underlying point scatters (figure 1(c)). Additional details about implementation are described by Zhang *et al* [7]. The current blind deconvolution code is scripted in MATLAB 2021b (MathWorks, MA, USA) and executed parallelly on a six-core Intel i7-8700K CPU. It takes about 54.9 min to process 7000 200 × 360 pixels CEUS images. One of the shortcomings of this approach, based on our prior experience [7], is that blind deconvolution is capable of distinguishing between closely located bubbles only when they are separated by more than one FWHM of the local PSF. For detecting micro blood vessels, this limitation has motivated the development of machine learning based techniques.

### 2.3. Generation of synthetic data for training and evaluation

**Figure 2.**
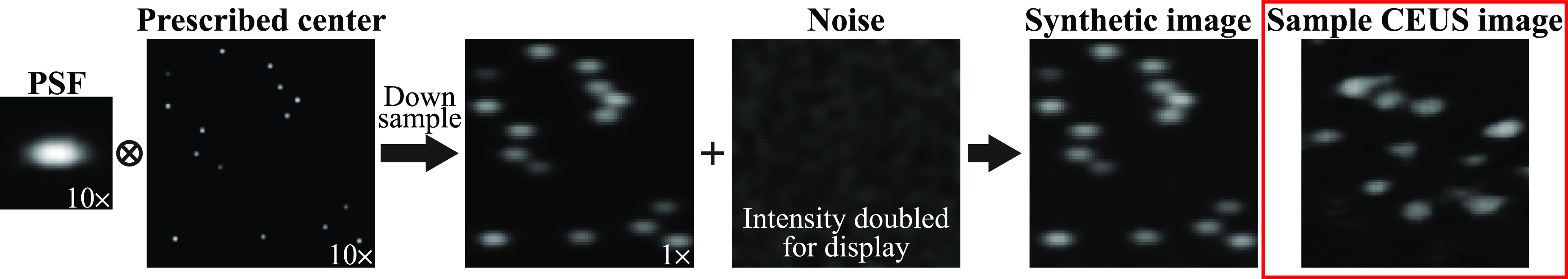
A sample procedure for generating the synthetic images. The subarea of a CEUS image containing a similar PSF is demonstrated next to the synthetic data.

**Figure 3.**
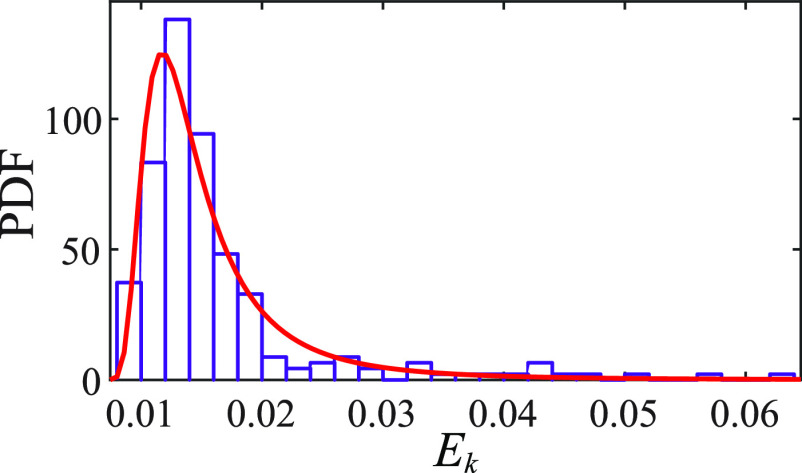
The PDF of the relative RMS intensity difference (*E_k_*) between the original PSFs and the up- and down- sampled ones.

The training and validation of the neural networks as well as the evaluation of different methods are conducted based on realistic synthetic images. This approach allows us to enhance the generalizability of the supervised networks for varying noise levels, bubble densities, and PSF morphologies with the exact knowledge of the locations of bubble centers. Several methods are available for generating realistic synthetic CEUS images of micro-bubbles, some of which simulate the propagation of acoustic waves through tissues [26], including a recent work that solves a modified Rayleigh–Plesset equation [40]. However, the current work involves CEUS images recorded using clinical scanners, which utilize unknown transmitted and received waveforms along with built-in algorithms for data pre- and post-processing. Consequently, we cannot rely on physics-based simulation tools to generate synthetic images that resemble the actual PSFs and noise distributions. Instead, we opt to use a phenomenological method, namely, estimate the intensity distributions of the PSFs and noise directly from the clinical CEUS images. Subsequently, following equation (1), the synthetic input training data are generated as a convolution of randomly distributed points (***x***) with the local PSF (***k***) along with added noise. As demonstrated in figure 2, ***x*** is generated on a high-resolution map in which the pixel size is one-tenth of that of the native CEUS image, i.e. it is comparable to the diameter of the contrast agent. The typical bubble concentration in the CEUS images obtained following the procedures in section 2.1 is about 0.5 mm^−2^, hence the synthetic bubble concentrations involved in the training vary between 0.1 and 1.0 mm^−2^. The image intensity varies from 0 to 1, each synthetic bubble center is assigned a random peak intensity in the 0.6–1 range to simulate the uneven bubble intensities in the actual CEUS images. The ***k*** originates from the spatially varying PSFs (figure 1(b)) obtained by blind deconvolution based on 15 different experimental datasets. These PSFs are further randomly rotated in the −20° to +20° range and resized using a factor ranging between 0.8 and 2. As illustrated in figure 2, ***k*** is also up-sampled to ten times the native image resolution using bicubic interpolation and then convolved with ***x***. The resulting image is subsequently down-sampled to the native image resolution using bicubic interpolation, forming an 80 × 80 pixel synthetic image, whose size would be 4.8 × 4.8 mm^2^. To evaluate the effects of up- and down-sampling on the intensity distributions of the PSFs, we up- and down-sample 228 PSFs to obtain $\boldsymbol{\hat k}$ and measure the root-mean-square (RMS) difference between the original (***k***) and processed ($\boldsymbol{\hat k}$) data. Figure 3 is the probability density function (PDF) of the relative RMS error, i.e.

\begin{align*}{E_{\boldsymbol{k}}} = RMS\left({\boldsymbol{\hat k}} - {\boldsymbol{k}}\right)/RMS\left( {\boldsymbol{k}} \right) \end{align*}

As is evident, the mode is about 1.2%, confirming that resizing of ***k*** does not introduce significant errors. The noise is modeled as low-intensity random speckles that are subsequently blurred by a 2D Gaussian filter. The PDFs of the noise intensity are obtained by sampling the background in the raw CEUS images and rounding their peak levels to 0.10, 0.15 (most commonly seen), and 0.20. In addition, we also generate a noisier artificial distribution by stretching the most intense PDF to 0.25. The example of noise and the final noisy synthetic image is shown in figure 2. For reference, we also provide a sample actual CEUS image with a similar PSF, showing that the synthetic data has similar characteristics.

To generate training references for the supervised learning methods (see sections 2.4 and 2.6), bubble center maps established at ten times the native image resolution have been downsized to three times the native resolution, respectively. After down-sampling, the pixels containing the true centers are prescribed values of 1, while others are set as 0. To improve training stability, the low-resolution center maps are blurred by a 5 × 5 2D Gaussian kernel, as recommended by Nehme *et al* [41]. In total, 250 000 input and reference image pairs have been created for the training and validation of supervised learning methods.

For evaluating the accuracy in detecting the bubble centers, the synthetic data involve four bubble concentrations, namely single bubble, as well as 0.25, 0.5, and 1 mm^−2^, corresponding to the typically observed low, normal, and dense bubble distributions. The five increasingly elongated PSFs along the scanline are based on experimental datasets, are not included in the training sets, and are free from additional random rotation and resizing. The peak noise levels are 0, 0.08, 0.16, and 0.24, and their PDFs are based on the CEUS data with a peak noise intensity of 0.15. A total of 2500 synthetic image pairs are generated for each bubble density, PSF, and noise level.

The same approach, but with modified PSFs, is used for generating the evaluation data for the SelfBD-net (see section 2.5). They are also based on five increasingly elongated PSFs that are not included in the previous training datasets and are further rotated by 90° to make them substantially different from any ones used above. For each PSF, 2500 image pairs with a bubble concentration of 0.5 mm^−2^ and a peak noise level of 0.16 have been generated without random PSF resizing. Moreover, synthetic images based on a particular 90°-rotated PSF with an aspect ratio of 1.81 are generated to evaluate the effects of peak noise levels ranging from 0 to 0.24, and bubble concentration ranging from 0.25 to 1 mm^−2^.

Our ultimate objective is to compare the ability of the different methods to identify closely separated microvessels and measure velocity profiles in macro vessels. Hence, we simplify the vessel complexity by using straight parallel vessel segments. In this way, the trends are directly related to the size, shape, and orientation of the PSFs relative to the vessel. Consequently, for evaluating the ability to distinguish between closely located microvessels, the synthetic bubbles are translated over 2500 time steps along two parallel lines separated by 18, 30, 60, 120, 180, 240, 300, and 360 μm at a typical mean microvascular velocity magnitude of 5 mm s^−1^. These lines are orientated either aligned or perpendicular to the major axes of the PSFs. New bubbles are generated based on the actual time history of microbubbles passing through a cross-section of a microvessel, where values based on different microvessels are assigned to each line. Moreover, to assess the accuracy of velocity measurements in macro blood vessels, the synthetic bubbles are propagated for 5000 time steps along tubular vessels with prescribed radii ranging from 120 to 720 μm. The prescribed velocity profiles are parabolic with a maximum value of 5 cm s^−1^ at the center. The vessels are also aligned in parallel and perpendicularly to the major axes of the PSFs. The bubble locations are randomly distributed with an average bubble distance of 330 μm, which is estimated based on the macro-vascular regions in actual CEUS data. For both micro- and macro- vascular evaluations, the PSFs are the same as those used for bubble localization evaluation and the noise profile has a peak noise intensity of 0.16.

### 2.4. SupBD-net

**Figure 4.**
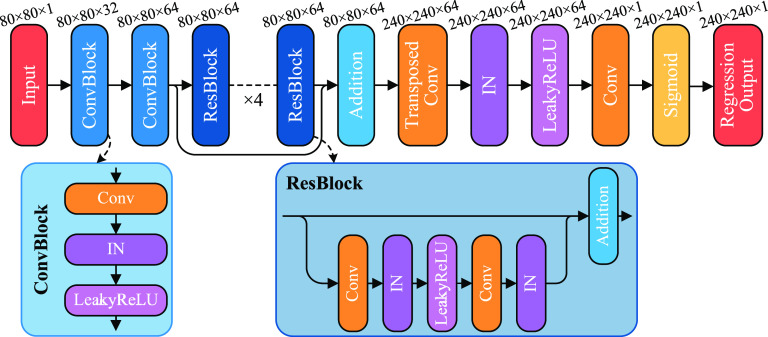
An illustration of the network structure of SupBD-net. The current SupBD-net consists of two ConvBlocks, four local residual blocks (ResBlock) along with one global residual block, one transpose convolution layer for upscaling, one final convolution layer for generating 2D images, and a sigmoid layer to ensure a 0–1 intensity range of the output image. The image size × number of feature channels are listed above each block.

As described in the introduction, we utilize a residual learning-based SupBD-net to achieve higher sub-pixel accuracy in localizing the bubble centers. The network structure mainly follows the previously developed super-resolution method SRResNet [33] but with some modifications tailored to the current application. Specifically, as illustrated in figure 4, the input raw CEUS images pass two convolution blocks for preprocessing and extracting shallow layer features. The components of each convolution block are illustrated in the bottom left of figure 4. Different from the typical elements, the ConvBlocks in SupBD-net consist of a 2D convolution layer followed by an instance normalization (IN) layer [42] and a leaky rectified linear unit layer (LeakyReLU). There are two reasons for this modification. First, IN has been proven to be favorable for single-image enhancement problems [43] by normalizing the mean value and covariance of each image in a minibatch. This step prevents the effects of instance-specific mean and covariance shift caused by variations in image intensity and noise level of specific data, hence enabling the network to focus on learning the common features of the trace-to-center conversion for every image. Second, the LeakyReLU layer performs a threshold operation, where any negative input is multiplied by a fixed scalar, selected as 0.2, following Radford *et al* [44], to prevent the assignment of zero gradients to negative values in ReLU. Subsequently, four residual blocks (ResBlock) are used for local residual learning, and the output is added to that of the second ConvBlock for global residual learning. Such a local-global residual learning scheme is adopted from a previous study of image super-resolution [32]. The number of local ResBlocks is selected based on a preliminary test, showing that four blocks achieve a good balance between localization accuracy and processing speed. The components of the ResBlock are sketched in the bottom right of figure 4. It has been modified from the typical residual block by replacing the batch normalization with IN and ReLU with LeakyReLU for the same reasons as previous steps. The features extracted by the local-global residual learning are up-sampled by a transposed convolution layer, which triples the spatial resolution. After another IN and LeakyReLU, a 2D convolution layer generates the high-resolution images. Finally, a sigmoid layer scales the data to ensure a 0–1 intensity range. It should be noted that, adopting the parameters used in SRResNet [33], the convolution kernel size is 9 × 9 for the first and last 2D convolution layers, and 3 × 3 for the others. The image size and the number of feature channels after each step in SupBD-net are listed above the corresponding block in figure 4.

The training of the SupBD-net is based on 250 000 pairs of synthetic images, out of which 70% are used for training and the rest as the validation set. An Adam optimizer [45] with a minibatch of 16 is used for training. For each epoch, the entire training dataset is randomly shuffled to bypass local optima, and the results are validated at the end of each epoch. The learning rate is initialized as 0.001 and is subsequently reduced by 10 for every 5 epochs [46]. The training ends when the validation loss stops decreasing over the next five epochs. The parameters of the trained network for the last five epochs are stored, and the network parameters giving the minimum validation loss are selected as the final result. The training is carried out on a desktop PC, equipped with an NVIDIA 1080Ti GPU, 1 Intel i7-8700K CPU, and 64GB DDR4 RAM. The training takes 116 h. For a trained SupBD-net, it takes 27.5 min to process 7000 200 × 360 pixels CEUS images.

### 2.5. SelfBD-net

As explained in the introduction, our objective in developing SelfBD-net is to establish an approach that is not limited to the scope of the training dataset, which can be generalized and applied to other datasets that have substantially different PSFs. Such a need is particularly important for CEUS images, where the bubble trace characteristics vary with the ultrasound wavelength, beamforming mechanism, transducer design, built-in post-processing algorithm, and the organ being imaged. The proposed SelfBD-net is derived from the conventional blind deconvolution method, where the cost function in equation (2) are separated as follows:

\begin{align*}{\mathcal{L}_{\boldsymbol{x, BD}}} = \underbrace {\left\| {\boldsymbol{x} \otimes \boldsymbol{k} - \boldsymbol{y}} \right\|_2^2}_{{\mathcal{L}_{\boldsymbol{y, BD}}}} + \underbrace {{\alpha _1}{{\left\| \boldsymbol{x} \right\|}_0} + {\alpha _2}{{\left\| {\nabla \boldsymbol{x}} \right\|}_0}}_{{\mathcal{R}_{\boldsymbol{x, BD}}}}\end{align*}

\begin{align*}{\mathcal{L}_{\boldsymbol{k, BD}}} = \underbrace {\left\| {\boldsymbol{x} \otimes \boldsymbol{k} - \boldsymbol{y}} \right\|_2^2}_{{\mathcal{L}_{\boldsymbol{y, BD}}}} + \underbrace {\gamma \left\| \boldsymbol{k} \right\|_2^2}_{{\mathcal{R}_{\boldsymbol{k, BD}}}}.\end{align*}

**Figure 5.**
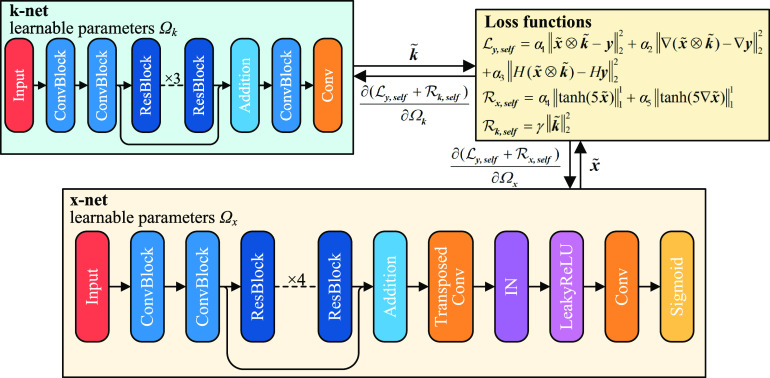
An illustration of the network structure of SelfBD-net. Shown in the top left is an additional network, named k-net, used for estimating the PSF from the raw CEUS image. The k-net and the pretrained x-net are jointly trained on the CEUS images with unknown PSFs using the loss functions provided in the top right.

In blind deconvolution, these equations are solved alternatively and iteratively for estimating the PSF and true centers. Here we introduce a self-supervised learning method that solves these optimization problems using neural networks. As illustrated in figure 5, in SelfBD-net, a new network, denoted as k-net, is added for estimating the PSFs, in addition to the center estimation network, which is denoted as x-net. The structure of the k-net follows that of the SupBD-net, except that there are only 3 local residual blocks, and there is no upsizing step nor final sigmoid layer. Also, after testing different sizes for the convolution kernels, we have selected a 7 × 7 kernel as a compromise between computation time and data quality. The currently selected x-net is the pretrained SupBD-net in order to take advantage of some of its learned center features, yet it is not limited to this selection.

**Figure 6.**
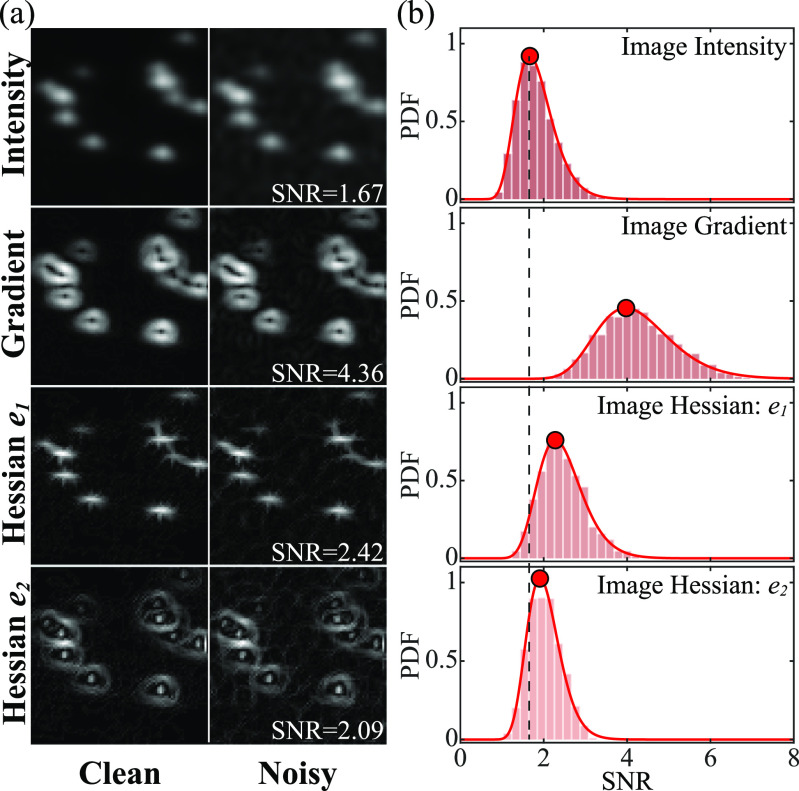
Comparison of the signal-to-noise ratio (SNR) for image intensity, gradient, and Hessian. (a) A sample comparison of a clean synthetic CEUS image (left) versus the same one with added noise at a typical level (right). From top to bottom, demonstrated images are image intensity, gradient magnitude, and the magnitudes of the first and second eigenvalues of the image Hessian. The corresponding SNR for the noisy image is listed in the bottom right of each image. (b) The SNR PDF for 2500 noisy synthetic images with a typical bubble density and noise level. The dashed line shows the center SNR level for image intensity, and the red dots show the center SNR level for each class.

The SelfBD-net is trained by solving cost functions that are based on equations (4) and (5), but with some modifications aimed at improving the results. As a starting point, the original loss term with the CEUS image ***y***, i.e. ${\mathcal{L}_{\boldsymbol{y, BD}}} = \left\| {\boldsymbol{x} \otimes \boldsymbol{k} - \boldsymbol{y}} \right\|_2^2$, is expanded to:

\begin{align*} {\mathcal{L}_{\boldsymbol{y, self}}}&amp; = {\alpha _1}\left\| {\boldsymbol{\tilde x} \otimes \boldsymbol{\tilde k} - \boldsymbol{y}} \right\|_2^2 + {\alpha _2}\left\| \nabla \left(\boldsymbol{\tilde x} \otimes \boldsymbol{\tilde k}\right) - \nabla \boldsymbol{y} \right\|_2^2\nonumber\\ &amp; \quad + {\alpha _3}\left\| {H\left(\boldsymbol{\tilde x} \otimes \boldsymbol{\tilde k}\right) - H\boldsymbol{y}} \right\|_2^2\end{align*}

by adding an *L*_2_ loss of the image gradient ($\nabla $) and Hessian (*H*) to the *L*_2_ loss of intensity. Here, $\boldsymbol{\tilde x}$ and $\boldsymbol{\tilde k}$ are the bubble centers and the PSFs estimated by the x-net and k-net, respectively. The weights, *α*_1_, *α*_2_, and *α*_3_, are selected as 1, 5, and 25 respectively, to ensure that each loss term is of the same order of magnitude as others. The two additional terms are introduced for the following reasons. First, the image gradient and Hessian have higher signal-to-noise ratios (SNRs) than image intensity. For example, figure 6(a) compares the image intensity, gradient, as well as the first (*e*_1_) and second (*e*_2_) eigenvalues of the Hessian matrix for a noise-free and noisy synthetic CEUS image. For each row, the SNR is defined as the RMS intensity of the clean image divided by the RMS of the intensity difference between the clean and noisy images. The results, indicated on each image, show that the gradient has the highest SNR, followed by the Hessians, and then by the image intensity. A more comprehensive comparison, which leads to the same conclusion, is provided in figure 6(b) by showing the PDFs of the SNR based on 2500 synthetic images with typical bubble densities and noise levels. Therefore, adding additional true information with higher SNR to the cost function is likely to improve the robustness of the SelfBD-net. Second, the eigenvalues of the image Hessian encode information about the shape and the orientation of the object in the image [47]. Hence incorporating this information into the cost function is likely to enhance the ability to obtain a PSF with the correct shape. It should be noted that, during implementation, the *L*_2_ loss is calculated directly for each term in the Hessian matrix instead of the eigenvalues, avoiding handling eigenvalues with complex numbers.

Next, the two *L*_0_ terms in equation (4) are non-convex and non-differentiable. Hence, as discussed by Wang *et al* [48], solving an *L*_0_ regularized optimization problem is computationally intractable, an issue known as non-deterministic polynomial-time hard [49]. In blind deconvolution, this issue is mitigated by introducing an approximate approach, which is detailed in Xu *et al* [50]. Alternatively, as a common remedy, other convex and continuous regularization terms, such as *L*_1_ or *L*_2_ norms, are used as a relaxation of the *L*_0_ norm. In the present SelfBD-net, we opt to use the *L*_1_ norms of $\tanh (5\boldsymbol{\tilde x})$ as a surrogate, which converts most of the non-zero values of $\boldsymbol{\tilde x}$ to almost 1. Hence, the sum of the absolute values of each element in $\tanh (5\boldsymbol{\tilde x})$ approximates the number of non-zero values in $\boldsymbol{\tilde x}$, i.e. the *L*_0_ norm. In this way, the two regularization terms for ***x*** can be approximated as:

\begin{align*}{\mathcal{R}_{\boldsymbol{x, self}}} = {\alpha _4}\left\| {\tanh \left(5\boldsymbol{\tilde x}\right)} \right\|_1^1 + {\alpha _5}\left\| {\tanh \left(5\nabla \boldsymbol{\tilde x}\right)} \right\|_1^1.\end{align*}

Following [39], and consistent with our previous experience with blind deconvolution, both *α*_4_ and *α*_5_ are selected as 4 × 10^−3^. As for the cost function for the k-net in SelfBD-net, which is equivalent to equation (5), the regularization term in equation (8) remains unchanged, with *γ* = 2, also following [39],

\begin{align*}{\mathcal{R}_{\boldsymbol{k, self}}} = \gamma \left\| {\boldsymbol{\tilde k}} \right\|_2^2.\end{align*}

Finally, as shown in figure 5, ${\mathcal{L}_{\boldsymbol{k, self}}} = {\mathcal{L}_{\boldsymbol{y, self}}} + {\mathcal{R}_{\boldsymbol{k, self}}}$ is used for training the k-net and ${\mathcal{L}_{\boldsymbol{x, self}}} = {\mathcal{L}_{\boldsymbol{y, self}}} + {\mathcal{R}_{\boldsymbol{x, self}}}$ for training the x-net.

**Figure 7.**
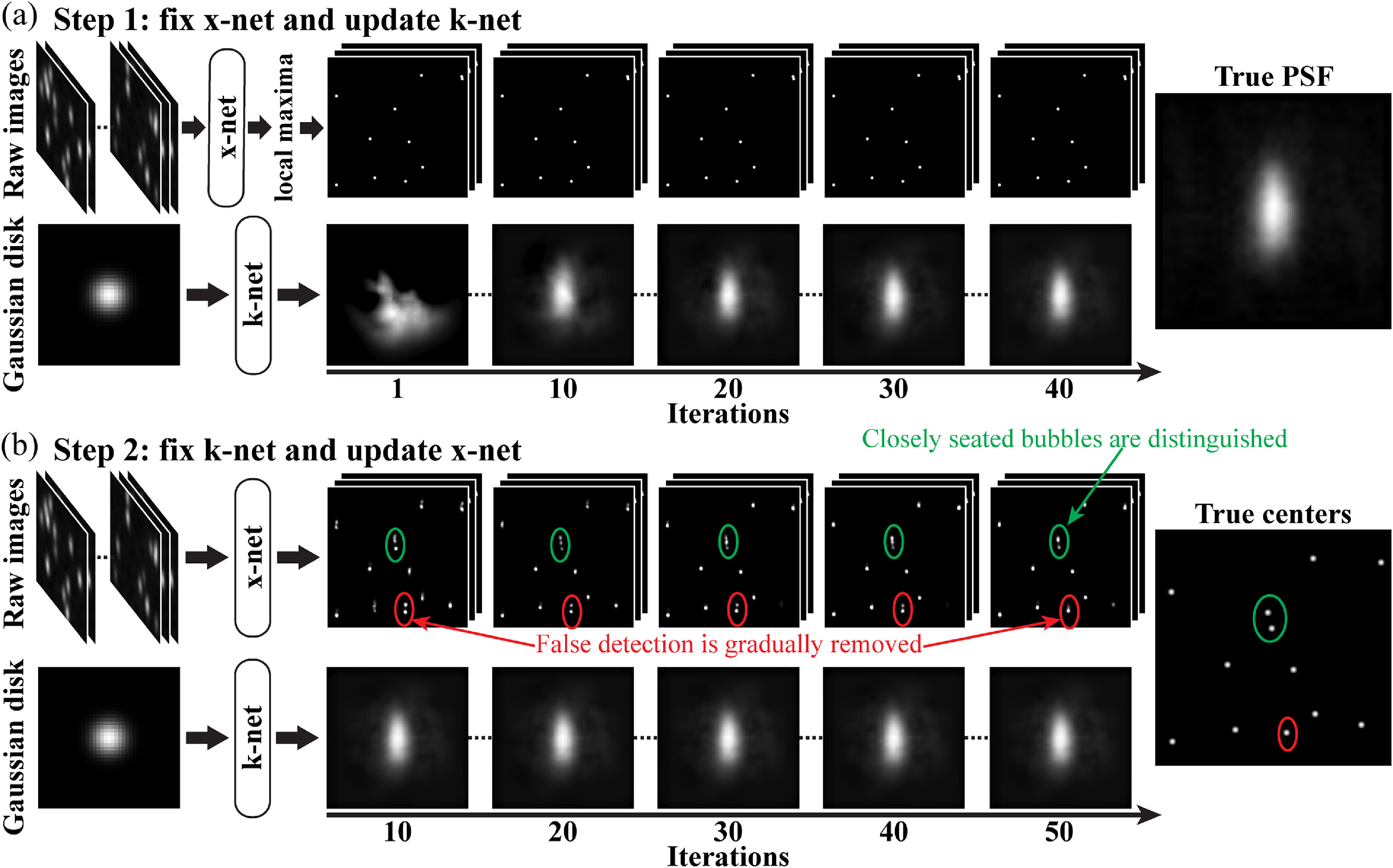
The procedures for training SelfBD-net. (a) Step 1: updating the k-net while freezing the x-net. After 40 iterations, the k-net converges and learns to deform the Gaussian disk to resemble the true PSF shown on the right. (b) Step 2: freezing the k-net and updating layers after the global residual learning in x-net. The first 50 iterations are demonstrated where the false detections (red circle) in the x-net output are gradually removed and the closely seated bubbles (green circle) are separated. The resulting estimated bubble centers are close to the true centers shown on the right.

SelfBD-net is implemented in each 80 × 80 interrogation window described in section 2.2. The training strategy depicted in figure 7 involves two steps: the first (figure 7(a)) is aimed at obtaining an estimation for the PSF. Hence, the parameters for the pretrained x-net are not updated, while the parameters of the k-net are randomly initialized and trained to deform an input Gaussian disk to the desired PSF. Since the image properties within the same 80 × 80 interrogation window are similar, for this step, only eight randomly selected images are fed into the x-net to generate the estimated $\boldsymbol{\tilde x}$ needed for calculating ${\mathcal{L}_{\boldsymbol{k, self}}}$. However, for an unknown PSF, the un-updated x-net is likely to yield false detections, with a particular tendency to generate multiple centers close to the true one. This issue, if left uncorrected, would lead to an underestimated PSF size and erroneous PSF intensity distribution. Hence, from each cluster of local maxima in $\boldsymbol{\tilde x}$, we select the brightest peak that exceeds 0.5 as the estimated location of the bubble center. The resulting sparse distribution (top row of figure 7(a)) is used for calculating ${\mathcal{L}_{\boldsymbol{k, self}}}$, while the corresponding true center map is presented on the right-hand side of figure 7(b). Note that since $\boldsymbol{\tilde x}$ could be of a higher spatial resolution than $\boldsymbol{\tilde k}$ and ***y***, to calculate ${\mathcal{L}_{\boldsymbol{y, self}}}$, the $\boldsymbol{\tilde k}$ is upsized to the same resolution as $\boldsymbol{\tilde x}$, convolved with $\boldsymbol{\tilde x}$, downsized to the native image resolution, and compared with ***y***. The sample training progress in figure 7(a) demonstrates the evolution of the estimated PSF in steps of ten iterations. The Adam optimizer is used for updating the k-net. The initial learning rate is 0.0001, and it is reduced by a factor of 10 when ${\mathcal{L}_{\boldsymbol{k, self}}}$ does not decrease for the next five iterations. The training is terminated when the RMS intensity difference in $\boldsymbol{\tilde k}$ between the current and the previous iteration is smaller than 1 × 10^−6^. The sample in figure 7(a) demonstrates that the k-net is able to gradually deform the input Gaussian disk to a shape that resembles the true PSF, which is shown on the right. For this particular example, the k-net output converges after about 40 iterations, which takes less than 19 s.

The next step, which is illustrated in figure 7(b), is to fix the k-net and refine the x-net. For each 80 × 80 interrogation window, eight randomly selected images are used for the validation, and the rest are used for training. For each iteration, a minibatch of eight random images is fed into the x-net and the resulting $\boldsymbol{\tilde x}$ is used for calculating ${\mathcal{L}_{\boldsymbol{x, self}}}$. Here, while the parameters of k-net are not updated, parameters for layers after the global residual block of the x-net are updated. The initial learning rate for x-net is 0.0001. For every five iterations, x-net is validated, and the learning rate is reduced by a factor of 10 when ${\mathcal{L}_{\boldsymbol{x, self}}}$ does not decrease for the next five validations. The reduction is repeated three times, and the network parameters yielding the least ${\mathcal{L}_{\boldsymbol{x, self}}}$ over the past five validations are selected as the final result. The first 50 iterations in this training procedure are demonstrated in figure 7(b). Initially, the x-net generates a bubble center map containing two or more false detections in the vicinity of each true bubble center. As the training goes on, the falsely detected items, e.g. those encircled in red, are gradually removed, and the green-encircled traces are eventually reduced to a pair of closely located bubbles. This refinement process typically takes about 90 s, and the resulting center map is very close to that of the true centers. Moreover, the updated parameters for the x-net of one interrogation window can be used for initializing those of its neighbors. Hence, the refinement durations for other windows can be further reduced. Once the SelfBD-net is trained for a new CEUS system, it takes 32.7 min to process 7000 200 × 360 pixels CEUS images.

### 2.6. Previously introduced deep learning based ULM methods

In addition to blind deconvolution (see section 2.2), the performances of SupBD-net and SelfBD-net are evaluated by comparing them to three recently introduced supervised learning methods, namely FCN-ULM [34], mDensenet-ULM [35], and Deep-ULM [25]. FCN-ULM utilizes a deep fully convolutional network consisting of two encoding levels and four decoding levels with transposed convolution. The mDensenet-ULM method [35] consists of eight densely skip connected blocks, each containing eight densely skip connected convolution layers. They are followed by two levels of transposed convolution. Finally, Deep-ULM is also an encoder-decoder based structure, but with 3 encoding levels, a latent level with 50% dropout, and 3 decoding levels with nearest-neighbor upsampling. In the current implementation of these methods, to establish fair comparisons, the output resolutions for all of them are set at *λ*/14, the same as the currently proposed techniques. Similarly, for blind deconvolution, after applying the deconvolution, the results are interpolated to a resolution of *λ*/14, before locating the bubble centers.

The training dataset, training methods, and the selection of training parameters of these networks are the same as those of section 2.4. The training takes 34, 72, and 37 h for FCN-ULM, mDensenet-ULM, and Deep-ULM, respectively. Once trained, it takes about 13, 24, and 14 min for each method to process 7000, 200 × 360 pixels, CEUS images.

## 3. Results and discussions

### 3.1. Bubble localization by supervised learning methods

**Figure 8.**
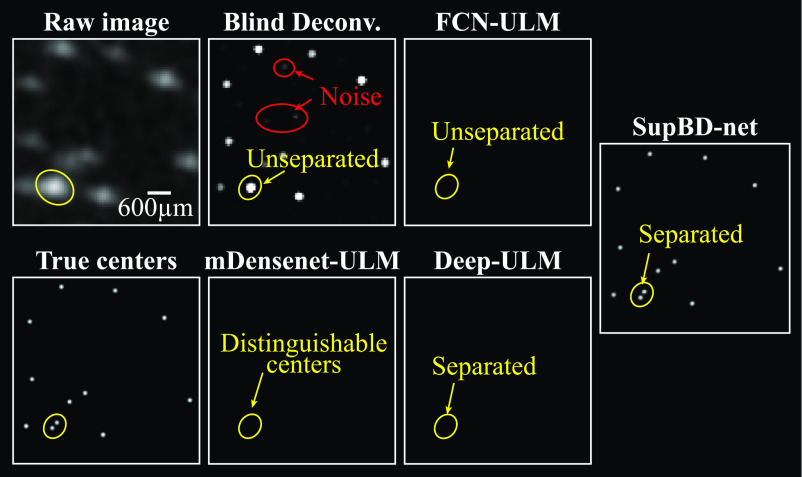
Sample images demonstrating the raw synthetic image, true bubble center map, and the center maps obtained based on blind deconvolution, FCN-ULM, mDensenet-ULM, Deep-ULM, and SupBD-net. The scale bar is provided at the bottom right of the raw image.

In this section, we evaluate the accuracies of bubble localization of FCN-ULM, mDensenet-ULM, Deep-ULM, and SupBD-net, and compare them with the conventional blind deconvolution. A sample raw synthetic image along with the corresponding true bubble centers and the center maps obtained by these methods are presented in figure 8. In the output of the blind deconvolution, the intensity distribution of each trace is quite uniform, introducing uncertainties in the intensity-weighted center location of bubbles, and limiting the ability to separate closely spaced ones. The bubble traces generated by the deep learning methods contain prominent peaks and dark edges, hence the bubble center location can be better estimated by intensity-weighted averaging. As shown in figure 8, for the pair of bubbles encircled in yellow, which are separated by 64 μm, blind deconvolution and FCN-ULM cannot separate them, and they are identified as a single bubble. While their traces slightly overlap in the mDensenet-ULM output, their centers can be identified. In the Deep-ULM and SupBD-net results, the two bubbles are clearly separated. Also, blind deconvolution is prone to enhance some of the noise, as illustrated by the areas encircled in red. This shortcoming could be mostly mitigated by setting an intensity threshold, e.g. selected as 1.5 times the mode of the histogram of the temporally averaged CEUS image intensity. Enhancement of noise is not an issue for the results obtained by deep learning methods.

**Figure 9.**
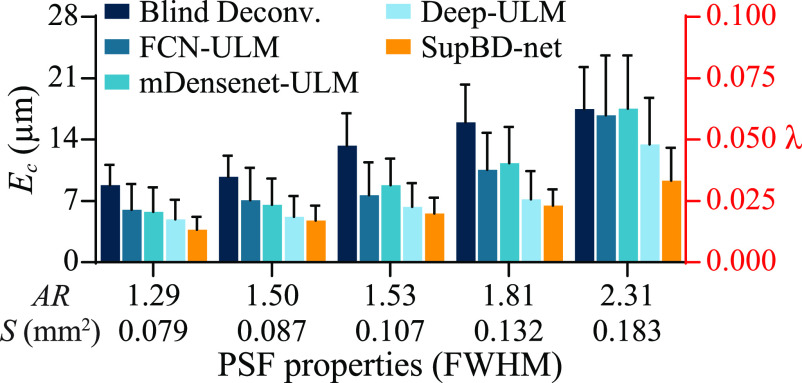
Variations of the mean error in the detected bubble center (*E_c_*—bars) and its standard deviation (error bars) with PSF area (*S*) and aspect ratio (*AR*) for a single bubble at a peak noise level of 0. The left vertical axis shows values in μm and the right shows values in fractions of wavelength (*λ*).

**Figure 10.**
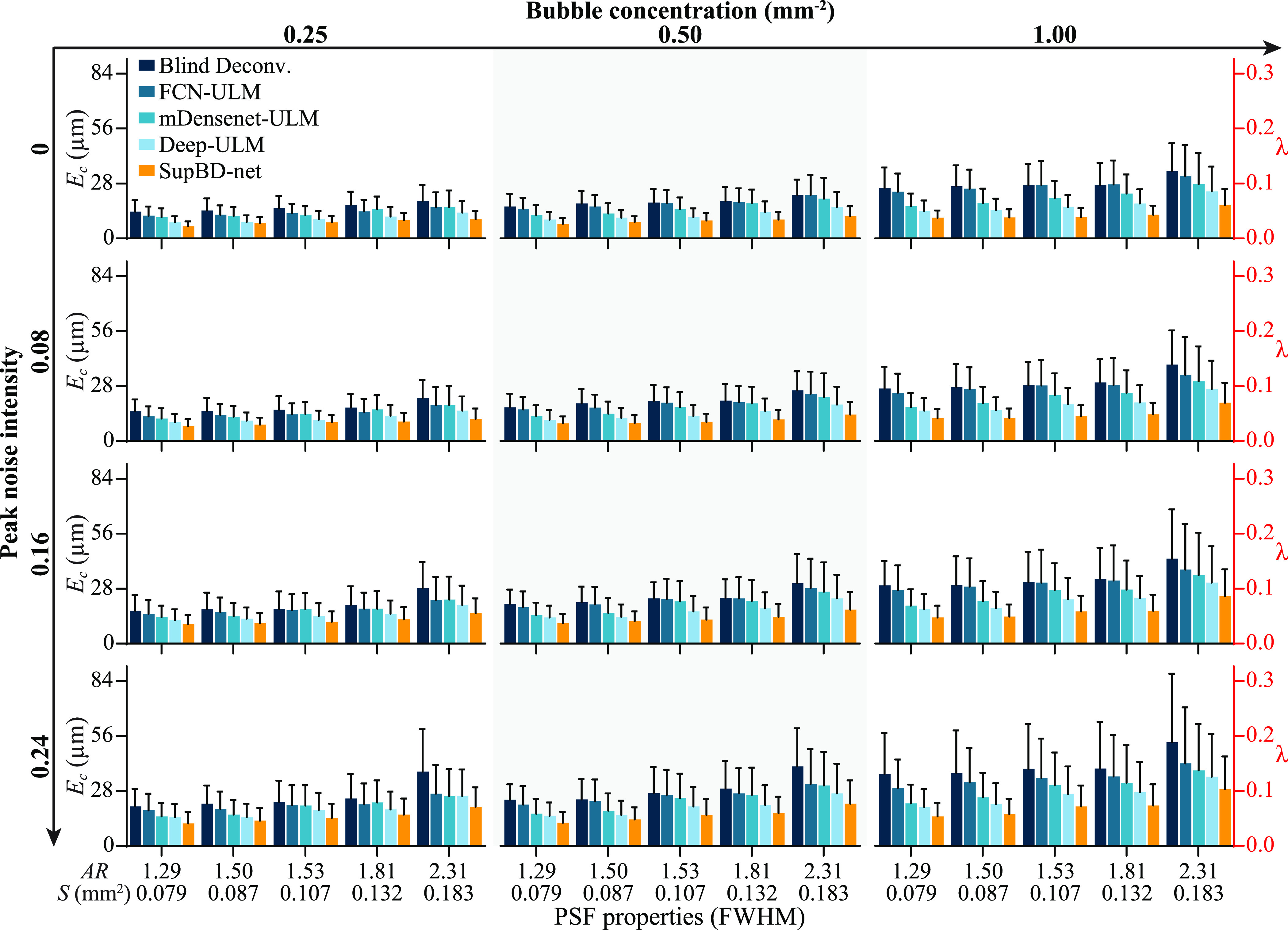
Variations of the mean error in the detected bubble center (*E_c_*—bars) and its standard deviation (error bars) for varying bubble concentrations (columns), peak noise levels (rows), and PSF area and aspect ratio (*S* and *AR*, horizontal axis). The left vertical axis shows values in μm and the right shows values in fractions of *λ*.

The accuracy of localizing bubble centers is quantified by the error in bubble center locations, *E_c_*, defined as the mean distance between the prescribed and the closest detected location of the bubble. For unseparated bubbles, where multiple true centers are detected as one, the distances are calculated for each true center. Starting from noise-free images of an isolated bubble, i.e. the most ideal case, figure 9 shows the effect of PSF area and aspect ratio, quantified based on FWHM, on *E_c_*, as well as its standard deviation (error bar) presented both in microns and fractions of *λ*. The errors increase with PSF size, as expected, with blind deconvolution having the largest errors (0.03–0.06 *λ*), and SupBD-net having the most accurate results, with errors ranging between 0.01 and 0.03 *λ*. The effects of noise and bubble concentration for images containing multiple bubbles are summarized in figure 10. Here, the columns, from left to right, show the effect of increasing bubble concentration, corresponding to the low (0.25 mm^−2^), normal (0.5 mm^−2^), and high (1.0 mm^−2^) levels. The rows, from top to bottom, show the effect of increasing peak noise levels, where 0.16 is commonly seen in experiments. In each panel, the errors of each method are presented for 5 PSFs with increasing area and aspect ratio. In general, for all cases, blind deconvolution yields the highest localization error, followed by the FCN-ULM, mDensenet-ULM, Deep-ULM, and then by SupBD-net. The variations of *E_c_* with PSF sizes, bubble concentrations, and noise levels are discussed as follows:

First, for the same bubble concentration and noise level, *E_c_* increases with the PSF size (area and aspect ratio). As a characteristic example, for normal concentration and typical noise level, *E_c_* for blind deconvolution increases from 20 to 31 μm with increasing PSF size, representing an uncertainty increasing from 0.07 to 0.11 *λ*. The corresponding FCN-ULM, mDensenet-ULM, Deep-ULM, and SupBD-net errors are 0.07–0.1, 0.05–0.1, 0.05–0.08, 0.04–0.06 *λ*, respectively. Hence, SupBD-net appears to be the least sensitive to the PSF sizes, followed by Deep-ULM, mDensenet-ULM, FCN-ULM, and then by blind deconvolution. Increasing the bubble concentration reduces the average nearest neighbor distance, hence the likelihood of overlapping traces and detection error increase. Consistent with the samples shown in figure 8, the SupBD-net method is the most successful in separating closely located bubbles, and is the least sensitive to the bubble concentration, resulting in the lowest localization uncertainties. Blind deconvolution is the least successful in separating traces, hence it is the most sensitive to the bubble concentration. Finally, as expected, for all methods, the uncertainty increases with the noise level. Yet, the changes in the SupBD-net errors are the lowest. For the highest bubble concentration and the largest PSF, the SupBD-net errors increase from 0.06 to 0.1 *λ* with the increasing noise level. The improved performance of SupBD-net over the other methods could be attributed to the following features of network design: First, compared to other network architectures, the presently utilized global and local residual learning have been proven by many recent state-of-the-art super-resolution methods [32, 51] to be particularly effective in resolving high-frequency details, which in our cases are the spatially distributed bubble centers. Second, residual learning mitigates the problem of vanishing gradients [29], enabling more effective training of deeper networks, hence capturing more complex patterns and details. Third, the batch normalization used in the other deep learning methods has been replaced by IN in SupBD-net. The latter is effective in removing instance-specific characteristics, ensuring that the model focuses on learning instance-invariant information rather than batch-level features [52]. Consequently, IN is better suited to handle input data with variations in PSF intensity distribution, bubble concentration, and background noise. Hence, SupBD-net achieves the highest accuracy in localizing the bubble centers and is the least sensitive to varying PSF sizes, noise levels, and bubble concentrations.

### 3.2. Bubble localization by the SelfBD-net

**Figure 11.**
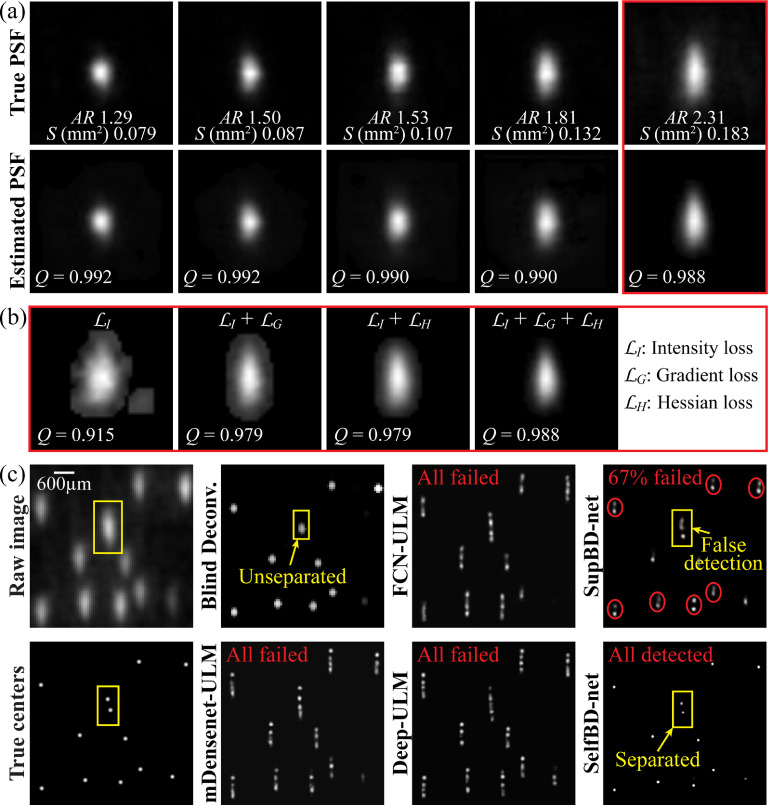
Visualization of the SelfBD-net outputs. (a) A comparison of the true (top row) and the estimated PSFs (bottom row) along with their correlation coefficients (*Q*). (b) A comparison of the right most PSF in (a) estimated using different loss functions. (c) A sample case comparing the estimated bubble centers of each method. Here, enclosed in the yellow box are two closely seated bubbles that are only correctly distinguished by the SelfBD-net.

Evaluation of SelfBD-net performance involves two aspects, namely the ability to reproduce the unknown PSFs and the errors in detecting bubble centers. Sample results are visualized in figure 11 using the synthetic images described in section 2.3, where the PSF is vertically aligned, i.e. perpendicular to the data used for training the supervised networks. The accuracy of reproducing the unknown PSF is quantified by the correlation coefficient (*Q*) between the intensity distributions of the true and the estimated PSFs. Images of the real and estimated PSFs are presented in figure 11(a), along with the corresponding values of *Q* for each sample. As is evident, the PSFs estimated by SelfBD-net are in good agreement with the real ones, with correlation coefficients varying between 0.988 and 0.992. The effectiveness of the SelfBD-net in identifying unknown PSF out of noisy CEUS images is attributed to the modified loss function, as defined in equation (6). This claim is supported by a comparison among the estimated PSFs obtained with different loss terms as demonstrated in figure 11(b). From left to right, the *Q* increases when the PSFs are estimated based on image intensity loss only, intensity + gradient loss, intensity + hessian loss, and finally the three loss terms combined. Such an improvement shows that while adding the image gradient and hessian loss separately could improve the accuracy of the PSF, these two terms contribute to different aspects of the improvement. As illustrated in figure 6, the image gradient loss term provides reference information that contains a much higher SNR, facilitating robustness against background noise. Whereas the hessian loss implicitly encodes the shape information of the PSF into the loss function [47], enhancing the ability of SelfBD-net to identify the intensity distribution of unknown PSFs. Hence, combining them further enhances the performance.

A sample set of images comparing the synthetic raw CEUS image along with its corresponding true centers to the center maps obtained by different methods is demonstrated in figure 11(c). Similar to the results in figure 8, blind deconvolution converts traces in the CEUS image into smaller blobs, which cannot distinguish closely located bubbles, e.g. the pair inside the yellow box. All the supervised learning methods encounter difficulties since the current PSF is substantially different from those of the training set. In fact, FCN-ULM, mDensenet-ULM, and Deep-ULM fail completely by treating the vertically oriented PSF as several vertically distributed bubbles. The same issue, but to a lesser extent, affects the SupBD-net outputs. Here, the elongated PSFs are interpreted as brighter centers with dimmer ones near them, both located close to the true center. Overall, 67% of the traces are interpreted as bubble pairs. In contrast, the centers estimated by SelfBD-net appear to be in good agreement with the true data, without false detections, and with the closely located centers successfully separated.

**Figure 12.**
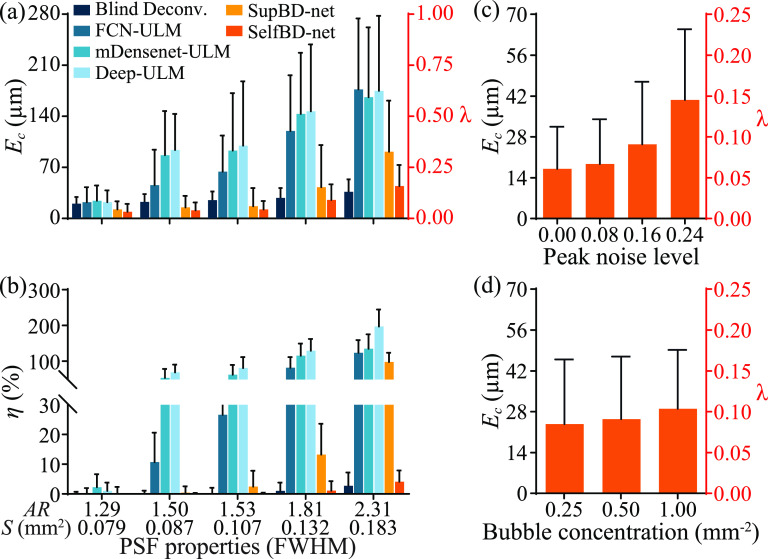
Evaluation of SelfBD-net using vertically oriented PSFs. Variations of *E_c_* (a) and the percentage of falsely detected bubbles (*η*, (b)) with increasingly elongated PSFs under a bubble concentration of 0.5 mm^−2^ and a peak noise level of 0.16. For the PSF with an aspect ratio of 1.81, also shown are the variations of *E_c_* with increasing noise level under a bubble concentration of 0.5 mm^−2^ (c) and with increasing bubble concentration under a peak noise of 0.16 (d). For (a), (c), and (d), the left vertical axis shows values in μm and the right in fractions of *λ*. For (a)–(d), the bar height shows mean values and the error bar shows the standard deviation.

Comparisons of *E_c_* based on synthetic data with a bubble concentration of 0.5 mm^−2^ and a peak noise level of 0.16, all for perpendicularly oriented PSFs, are presented in figure 12(a). In cases of falsely detecting pairs/clusters of bubbles (figure 11(c)), all the distances to the true center are accounted for. The corresponding percentages of falsely detected bubbles *η* are shown in figure 12(b). Using the PSF with an aspect ratio of 1.81 as an example, figures 12(c) and (d) show the effects of noise and bubble concentrations on the localization errors of SelfBD-net, respectively. Figure 12(a) indicates that the SelfBD-net errors are significantly lower than those of the other methods, remaining below 0.15 *λ* for all the PSFs. The blind deconvolution errors increase mildly with the aspect ratio but remain in the 0.07 to 0.13 *λ* range. Such values are also consistent with the blind deconvolution results of the horizontally oriented PSFs with the same bubble concentration and noise level in the previous section. The errors of FCN-ULM, mDensenet-ULM, and Deep-ULM increase rapidly as the aspect ratio reaches 1.50, and those of the SupBD-net increase significantly for aspect ratio larger than 1.81. The fraction of false detections (figure 12(b)) of SelfBD-net remains less than 4% for all the PSFs. Consistent with the sample in figure 11(c), false detection of multiple bubbles is not an issue for blind deconvolution. For aspect ratios smaller than or equal to 1.50, all supervised learning methods are able to handle unfamiliar PSFs with false detections of less than 5%. However, they fail at higher aspect ratios, with SupBD-net above 1.81, and others at 1.50 or higher. Hence, the current training strategies for supervised learning methods are able to extend their generalizability but to a limited extent. While the SelfBD-net errors increase with noise level for a bubble concentration of 0.5 mm^−2^ (figure 12(c)), they remain well below 0.15 *λ*. Furthermore, for a characteristic noise level of 0.16 (figure 12(d)), *E_c_* does not increase significantly with the bubble concentration. Clearly, for the perpendicular PSFs, i.e. cases that the supervised learning methods are not trained for, the performance of SelfBD-net is superior to that of the other techniques, giving the least errors and false detections. This performance is attributed to the training scheme of SelfBD-net, including: (i) the phenomenological model of the CEUS image, i.e. equation (6); (ii) the strong constraints on the sparsity of the bubble center distribution, i.e. equation (7); and (iii) the well estimated PSF (figure 11). These tools give SelfBD-net better generalizability than that of other supervised learning methods.

**Figure 13.**
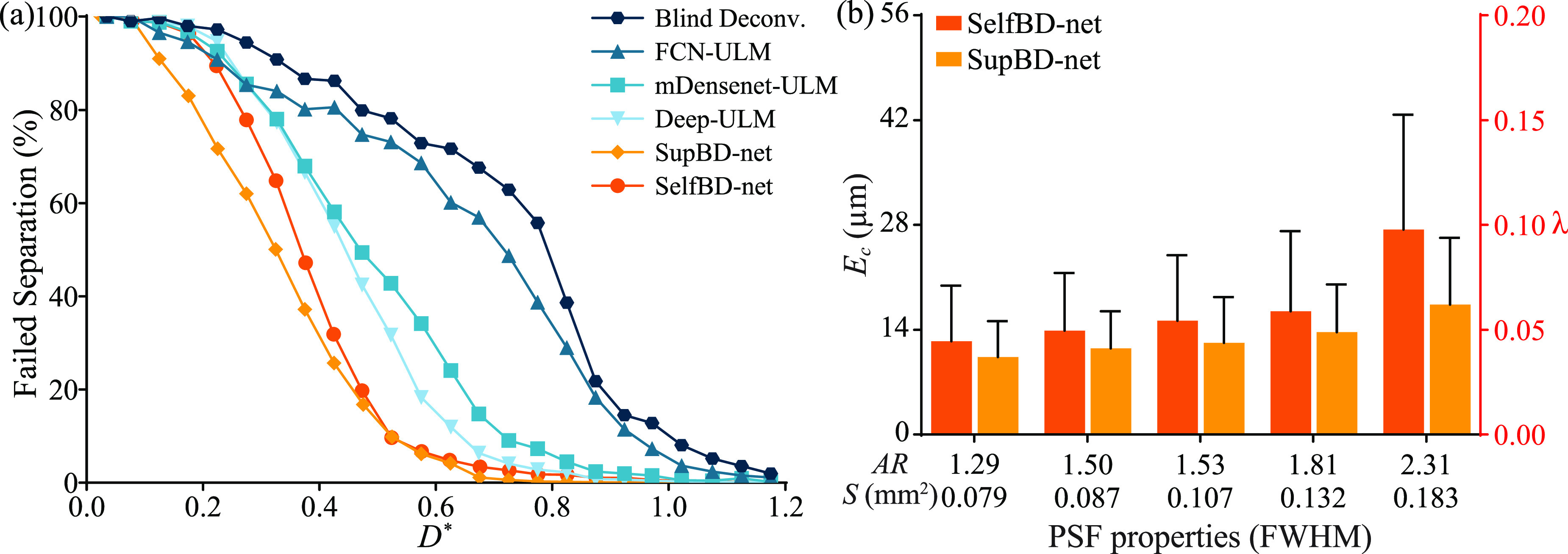
Evaluation of SelfBD-net using the horizontally oriented PSFs with a bubble concentration of 0.5 mm^−2^ and a peak noise level of 0.16. (a) Variations of the percentage of unseparated bubbles with the bubble distance normalized by the PSF FWHM length along the line connecting the two centers (D*). (b) Comparison of *E_c_* between SelfBD-net and SupBD-net for increasing PSF size. The left vertical axis shows values in μm and the right in fractions of *λ*. The bar height shows mean values and the error bar shows the standard deviation.

Figure 13 compares the performance of SelfBD-net using the evaluation dataset described in section 3.1, i.e. images with horizontally oriented PSFs. The analysis focuses on the case with a bubble concentration of 0.5 mm^−2^ and a peak noise level of 0.16. The first part of the comparison examines the ability to distinguish closely located bubbles with overlapped bubble traces. The data are grouped in terms of D*, the center-to-center distance of each bubble from its nearest neighbor normalized by the length of the PSF FWHM along the line connecting the two centers. The percentage of unseparated bubble traces as a function of D* is plotted in figure 13(a). As is evident, in all cases, all the methods fail at some D*. Blind deconvolution has the worst performance, with the fraction of failed separation exceeding 5% at D* < 1.05, closely followed by FCN-ULM (D* < 1.0), then with improved outcomes by mDensenet-ULM and Deep-ULM (D* < 0.8 and 0.7, respectively), and finally SelfBD-net and SupBD-net (D* < 0.6). While the difference between the latter two is small, as expected for this dataset, SupBD-net performs better for D^*^ < 0.5. The effect of PSF size on *E_c_* for SelfBD-net and SupBD-net are plotted in figure 13(b). Results for the other methods, which have higher errors are already presented in figure 10. Both techniques maintain subpixel errors ranging between 0.05 and 0.1 *λ*, with those of SelfBD-net being slightly higher. This difference can be traced back to the effect of noise. While SupBD-net is trained by directly associating the noisy data to the true centers, SelfBD-net only partially accounts for the noise by adding terms with higher SNR. Hence, even with correctly estimated $\boldsymbol{\tilde x}$ and $\boldsymbol{\tilde k}$, the noise in ***y*** would result in a non-zero loss in equation (6), hence the optimizer would adjust the parameters of the x-net and k-net that introduce errors in $\boldsymbol{\tilde x}$ and $\boldsymbol{\tilde k}$. Therefore, although SelfBD-net is initiated based on the bubble centers estimated by SupBD-net, its output may deviate slightly after a few iterations.

### 3.3. Spatial resolution of micro-vessel detection

In this section, we evaluate the ability of the various image processing methods to distinguish closely located parallel bubble trajectories, and to determine the errors in their locations for varying PSF sizes and distances between them (*D_l_*). As discussed by several recent works, e.g. [11, 19], these trajectories are used for mapping the location of micro- and macro-blood vessels in internal organs. Note that the bubbles are generated and translated along the lines at two series of independent time points, where bubbles from each line could be overlapped or isolated at different time steps. The PSFs are aligned horizontally, and the vessels are either horizontal (figure 15(a)) or vertical (figure 15(b)). Hence, the PSF dimension that affects the ability to resolve closely spaced vessels is the FWHM length in the direction perpendicular to the vessel (*L_p_*), as indicated in the figures. The resulting bubble trajectories are pixelated on images at 10 times the native image resolution, where the pixel size is equivalent to the microbubble diameter.

**Figure 14.**
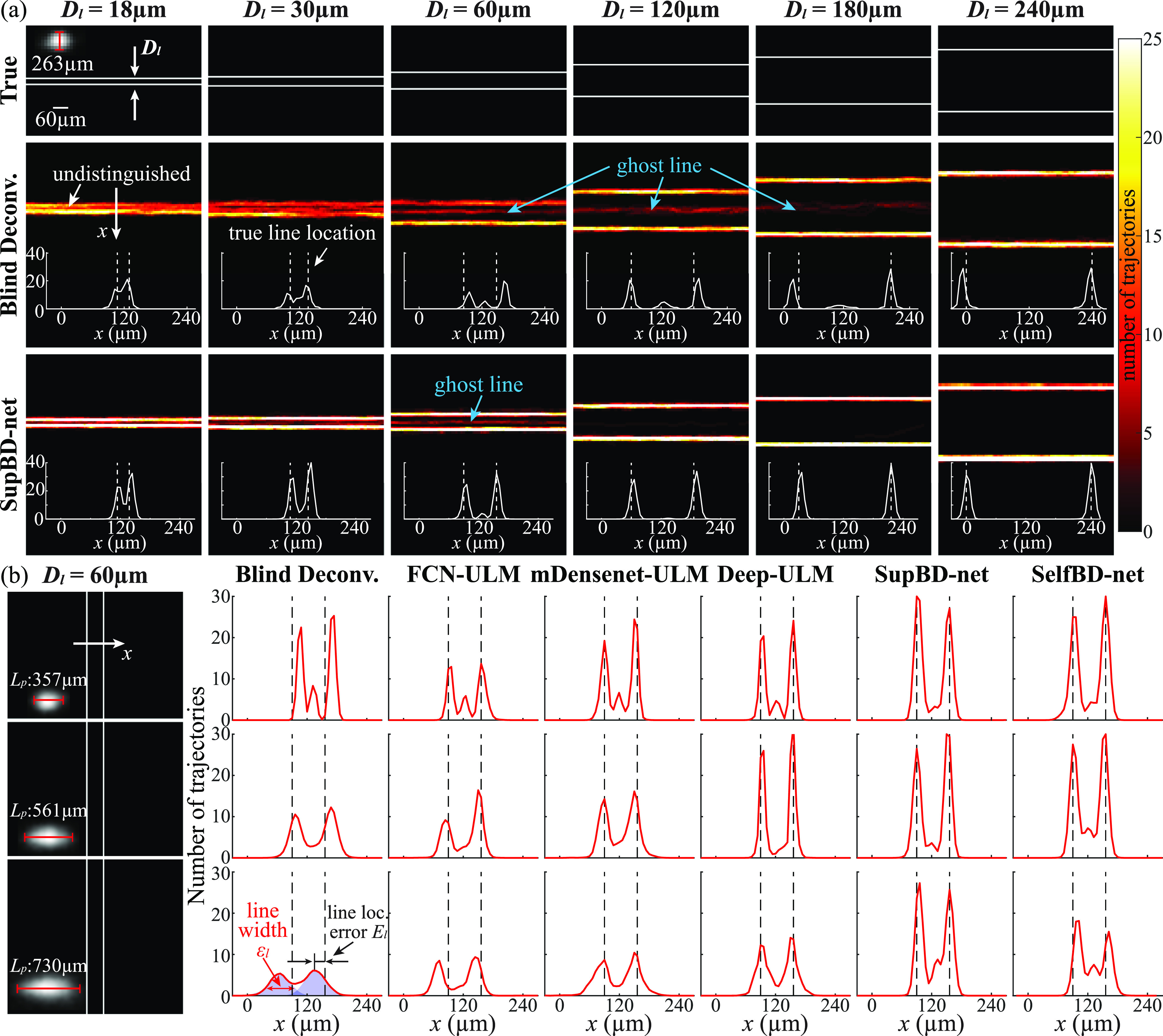
Spatial resolution of closely located parallel lines for different image processing methods. (a) Sample heat maps of bubble trajectories showing the effect of increasing line spacing (*D_l_*). (b) Sample cross-sectional profiles averaged along the line showing the effect of PSF FWHM length in the direction perpendicular to the line (*L_p_*). The error in line location (*E_l_*) and line width (*ϵ_l_*) are indicated in the bottom left. For (a) and (b), the vertical dashed lines show the true location of each line. The PSF size is not shown on the same scale as the line spacing.

Figure 14(a) demonstrates the impact of *D_l_* on the detected pair of horizontal vessels for a fixed *L_p_* of 263 μm. The reference line spacing is shown in the top row, the blind deconvolution results are presented in the middle row, and the SupBD-net data are in the bottom row. For both cases, the reconstructed lines and their cross-sectional profiles averaged along the line are provided. Also, each profile plot shows the location of the original line, and its distance from the detected peak represents a location error, which is quantified later. Figure 14(a) shows that for blind deconvolution results at *D_l_* = 18 μm, the two lines cannot be distinguished. As *D_l_* increases to 30 μm, the two lines are separated, but with considerable overlap, and connected tails. For *D_l_* = 60 μm, the image contains two primary lines with a ‘ghost’ line between them. The latter occurs in cases where two bubbles appear at nearly the same time, and consequently overlap, resulting in trajectories defined by the combined PSF. The intensity of the ghost line diminishes with increasing line spacing and disappears for *D_l_* = 240 μm. The existence of a ghost line poses a challenge since one cannot simply dismiss it in a real image where the number of bubble traces varies among vessels. This topic is discussed later, as we introduce a systemic method for determining the proper threshold to identify a vessel. A similar progression that includes two overlapped lines, the formation of a ghost line, and complete separation occurs also for the SupBD-net data. Here, the two lines are already detected at *D_l_* = 18 μm, and while the ghost line appears at 60 μm, it completely disappears for *D_l_* ⩾ 120 μm. Moreover, it could be observed that the blind deconvolution results have larger errors in the line location, especially for small line spacings. In contrast, the detected line location is very close to the reference location for all line spacings in SupBD-net results. The effect of PSF size is demonstrated for all four techniques in figure 14(b) using examples of vertical trajectories for a fixed *D_l_* of 60 μm. For all cases, *L_p_* is much larger than the line spacing. In general, for each *L_p_*, the intensity of the ghost line is the highest for blind deconvolution, followed by FCN-ULM, mDensenet-ULM, Deep-ULM, SelfBD-net, and SupBD-net. For all techniques, with increasing *L_p_*, the intensity of the ghost line diminishes as it merges with the tails of the two main peaks. These trends are consistent with the ability to separate closely located bubbles, as demonstrated in figure 13(a). Moreover, the line location errors also increase with increasing *L_p_* for all methods, where errors are larger in blind deconvolution and FCN-ULM results than those of other techniques. Clearly, the improved accuracy in locating the bubble centers by SupBD-net has led to improved detection of closely located microvessels.

**Figure 15.**
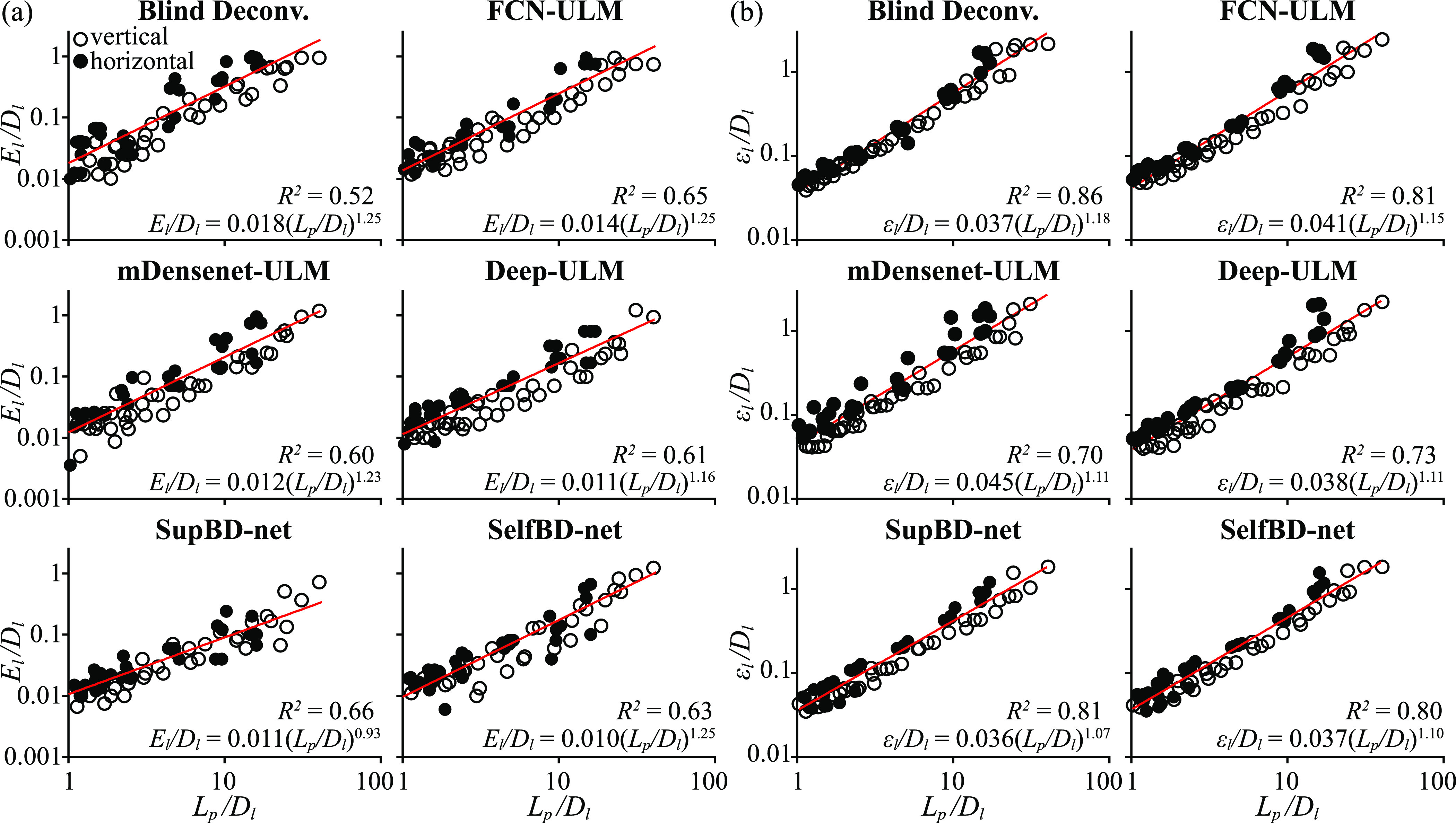
Variations of nondimensionalized: (a) line location error (*E_l_/D_l_*), and (b) line width (*ϵ_l_/D_l_*) with PSF size (*L_p_/D_l_*). The red line shows the least-square-fitted power law, whose expression as well as the coefficient of determination (*R* ^2^) are provided at the bottom of each panel. The solid dots denote data of horizontal lines, and hollow dots represent data of vertical lines.

The next discussion quantifies the uncertainty in the location of trajectories. As illustrated for the profile presented in the bottom left of figure 14(b), each profile is fitted with the sum of multiple Gaussian distributions (shadowed region) that match the number of peaks. This procedure is performed using the MATLAB Curve Fitting Toolbox. The line location error (*E_l_*) is defined as the distance between the primary Gaussian peaks and the corresponding true line location. The measured line width (*ϵ_l_*) is defined as twice the standard deviation of each fit. Since the spatial resolution of the parallel lines is affected by both *D_l_* and *L_p_*, we plot in log scales in figure 15 the values of *E_l_/D_l_* and *ϵ_l_/D_l_* vs. *L_p_*/*D_l_*. Figure 15(a) shows that for all methods, when *L_p_/D_l_* is in the order of one, all the errors decrease to 0.01–0.02 *D_l_*, i.e. results are very accurate. With increasing *L_p_/D_l_*, the increase in *E_l_/D_l_* appears to follow a power law. The exponent, which is indicated on each graph, is the highest for blind deconvolution, FCN-ULM, and SelfBD-net, followed by mDensenet-ULM and Deep-ULM, and then SupBD-net, which has the lowest errors for high *L_p_/D_l_*. All the coefficients have a similar order of magnitude, with the blind deconvolution values being 60% higher than those of SupBD-net and SelfBD-net. The nondimensionalized line width (figure 15(b)) also shows a power law relation with *L_p_/D_l_*, with the least exponent and coefficient obtained for SupBD-net. Also, trends are better aligned with the line fits than those of *E_l_/D_l_*, as quantified by the higher coefficient of determination (*R* ^2^) indicated on each graph.

**Figure 16.**
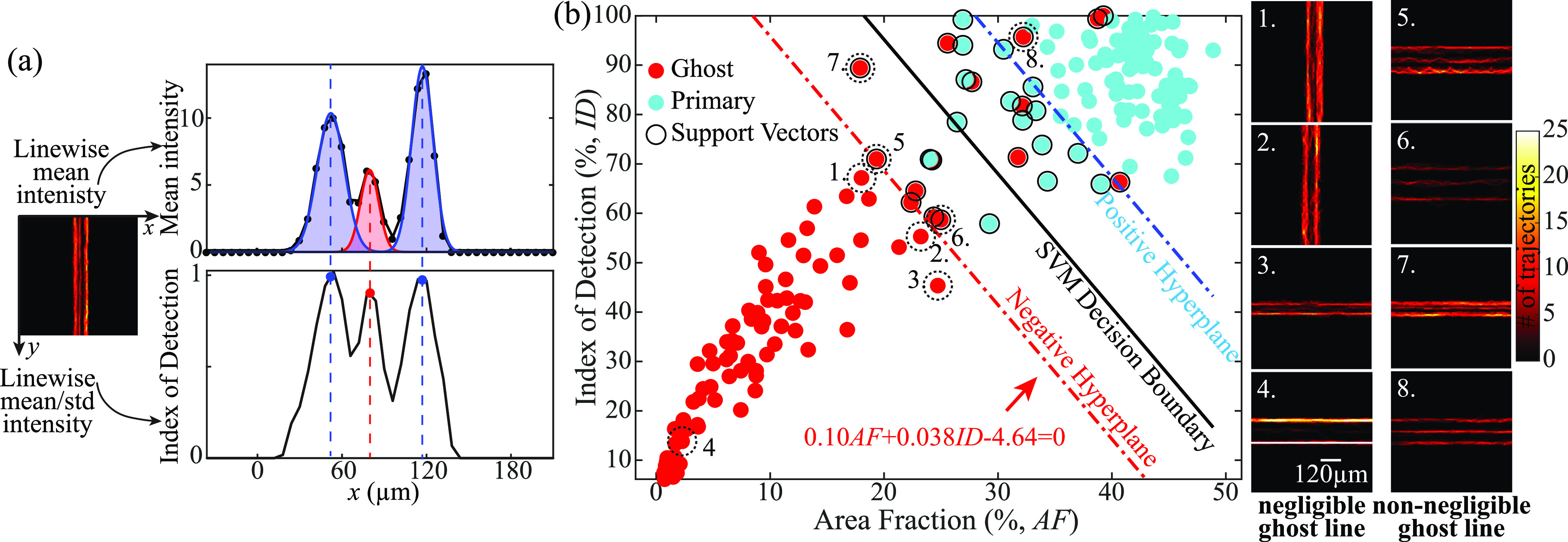
Determining the threshold for negligible ghost lines using support vector machine (SVM). (a) Sample illustration of the definition of the parameters, namely, the area fraction, *AF*, and the normalized index of detection, *ID*. (b) Scatter plots of *AF* and *ID*, as well as the decision boundaries estimated by the SVM. The red dots correspond to ghost lines, the blue dots to primary lines, and the black encircled ones are the support vectors. A few examples are shown on the right side demonstrating cases with negligible ghost lines (cases 1–4), and non-negligible ones (cases 5–8).

Returning to the ghost line (figure 14), its existence might cause false detection, where two closely located microvessels are identified as three. While some ghost lines share similar features as the primary ones, e.g. the blind deconvolution results for *D_l_* = 60 μm, others are dimmer and discontinuous, e.g. the SupBD-net results for *D_l_* = 60 μm, hence they can be identified and filtered out. Two parameters have been selected to distinguish the ghost lines from the primary ones. As illustrated in figure 16(a), the first is the area fraction (*AF*) defined as the area under each line divided by the total area covered by all the lines. The value of *AF* associated with the ghost line quantifies the percentage of falsely detected bubbles. To calculate *AF*, the linewise averaged heatmap profiles are fitted with the sum of multiple Gaussian profiles, and the areas are calculated based on these fits. The second parameter is the index of detection (*ID*), defined for each line as the peak linewise average intensity divided by the linewise standard deviation at the same location. Since the magnitude of *ID* varies, for each case, we normalize the result by the maximum value. A sample normalized profile of *ID* is provided in the bottom plot of figure 16(a). This parameter quantifies the structural continuity along the line, where dim structures with low means or discontinuous structures with high standard deviations have low *ID*s. A scatter plot of *AF* and *ID* is presented in figure 16(b), where red symbols correspond to ghost lines and blue symbols to the primary lines. As is evident, the data are generally divided into two groups, but with some ambiguities in the middle. To determine a threshold for separating these two groups, we utilize a support vector machine (SVM), provided by MATLAB Statistics and Machine Learning Toolbox, to search for an optimal decision boundary that separates the data into two classes [53]. In figure 16(b), the black encircled dots are the support vectors, the black line shows the decision boundary, and the red and blue lines show the negative and positive hyperplanes, respectively. A few examples are demonstrated on the right to visualize the effectiveness of this approach. Close to the negative hyperplane, the ghost lines appear to be visually negligible for cases located to the left, e.g. cases 1–3, whereas those on the right, e.g. cases 5–7, appear to be similar to the primary lines. Far below the negative hyperplane, e.g. case 4, there is no visible ghost line. In contrast, on the right of the positive hyperplane (case 8), the ghost line is similar to the primary lines. Hence, we have selected the negative hyperplane as the boundary of negligible ghost lines. Its functional relationship is:

\begin{align*}0.10AF + 0.038ID - 4.64 = 0 \end{align*}

**Figure 17.**
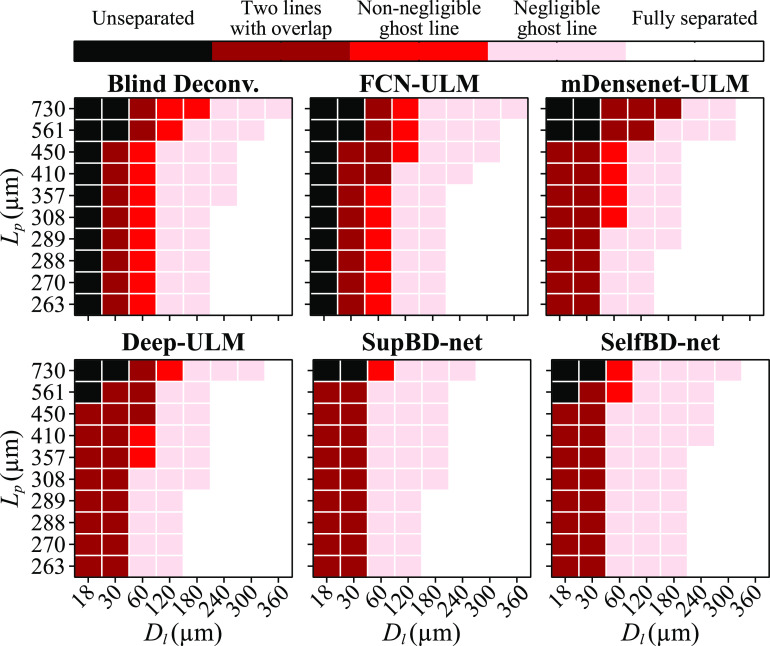
Summarization of the ability of each method to separate closely located parallel lines for different *D_l_* and *L_p_.*

Based on this approach, we have divided the outcome of each method into five categories, namely, unseparated lines, separated into two lines with overlap, separated with non-negligible ghost lines, separated with negligible ghost lines, and fully separated. For two lines with significant overlap, if the intensity of the trough between them is larger than 95% of the primary line intensity, these two lines are considered unseparated. The distinction between non-negligible and negligible ghost lines is defined based on the SVM analysis and equation (9). The results are summarized in figure 17. It shows that in all cases blind deconvolution and FCN-ULM are unable to resolve lines that are 18 μm apart, and when the PSF size is larger than 561 μm, they cannot resolve lines that are 30 μm apart. For most lines that are 60 μm apart, blind deconvolution yields ghost lines that are non-negligible compared to the primary lines, but as the line spacing increases from 120 to 360 μm, most of the cases are either separated with negligible ghost lines or fully separated. Among the various techniques, blind deconvolution and FCN-ULM have the largest fraction of cases with unseparated lines, followed by mDensenet-ULM, and then Deep-ULM and SelfBD-net, with SupBD-net having the least fraction. A reversed trend is evident for the fraction of negligible ghost lines and fully separated ones. Clearly, SupBD-net is the most effective, achieving clear separation even when *D_l_* is only 11% of the PSF size.

It should be noted that the current analysis is limited to two parallel horizontal and vertical lines. In actual CEUS images, the microvessels are aligned in all directions and there are usually more than two microvessels in the vicinity of each one. Hence, as demonstrated in section 3.5, the separation of microvessels in a real system is more challenging. Nonetheless, the present analysis provides a valuable comparison between the six techniques.

### 3.4. Flow measurement for macro-vessels

**Figure 18.**
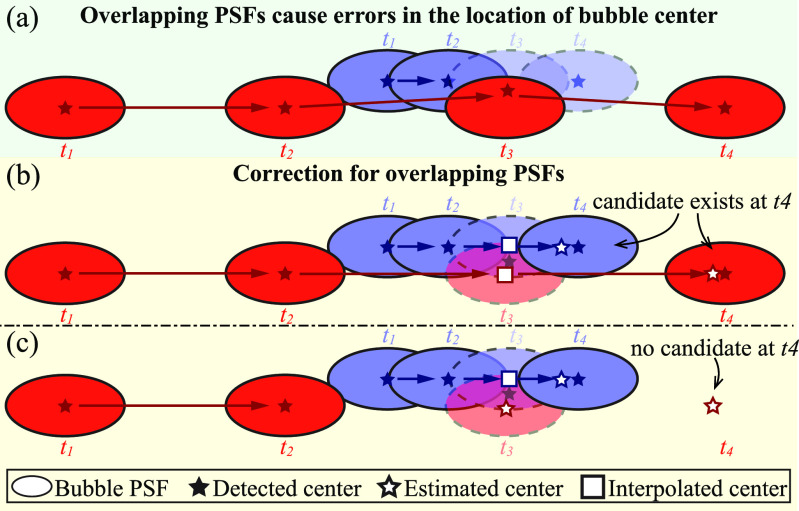
Correction of the error in velocity measurement caused by falsely detected bubble centers of overlapping PSFs. (a) The bubble-to-track assignment without correction for the overlapping PSFs. Overlapping PSFs correction strategies when a candidate is (b) detected (solid star) and (c) not detected in the vicinity of estimated (hollow star) bubble location at *t*_4_. For each track, once a candidate is assigned at *t*_4_, the location for the overlapping PSF at *t*_3_ is replaced by the interpolated value (hollow box). Otherwise, it is terminated at *t*_2_.

**Figure 19.**
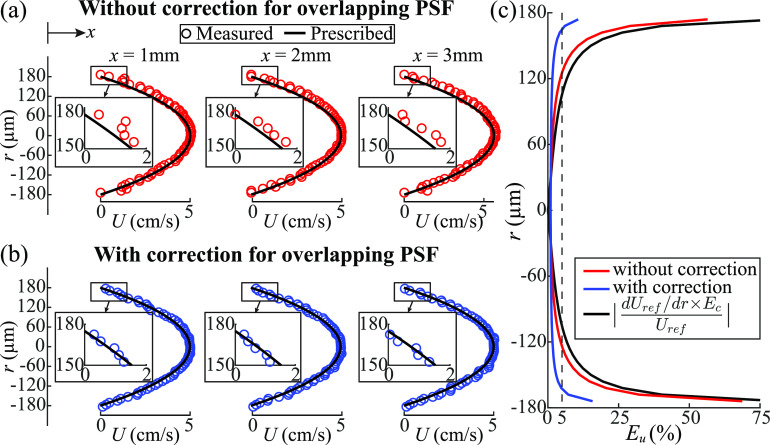
(a) Sample SupBD-net results showing velocity profiles at three streamwise locations of bubbles (*L_p_* = 263 μm) traveling inside a horizontal blood vessel (*R*= 180 μm) measured without correction for overlapping PSFs. The measured values are marked by circles and the prescribed velocity profile is plotted in curves. (b) The corresponding velocity profiles measured with correction. (c) The relative error in velocity (*E_u_*) measured without (red) and with (blue) correction for the overlapping PSF. Also plotted in black is *E_u_* caused by the error in bubble center detection, i.e. $\left| {(dU{_{ref}}/dr \times {E_c})/{U_{ref}}} \right|$.

This section evaluates the ability of various methods to determine the velocity distributions in large blood vessels, especially for cases where the vessel diameter is of the same order as the PSF. The primary challenges involve errors in the location of bubble centers in regions with significant velocity gradients, such as near a vessel wall for cases with a parabolic velocity profile. For example, figure 18(a) shows two bubble tracks with different velocities moving in parallel, with the detected centers indicated by stars. At *t*_3_, the slower and faster bubble traces partially overlap leading to the false detection of a single center between the overlapping PSFs. The conditions for such a false detection are discussed in previous sections. Consequently, without additional procedures to correct the trajectories, the detected center is assigned to the faster track because of the lower relative error in velocity, while the slower track is terminated at *t*_2_. This process would predict a velocity higher than the locally prescribed value in the vicinity of the false center. To quantify the resulting errors in measuring the velocity profile in blood vessels, we have performed the following analysis based on synthetic images. The synthetic data are created by propagating bubbles along tubular vessels with prescribed radii ranging from 120 to 720 μm, where the velocity profiles are parabolic with a maximum value of 5 cm s^−1^ at the center of the tube. The vessels are aligned in parallel and perpendicularly to the major axes of the PSFs. The bubble locations are randomly distributed with an average bubble distance of 330 μm. The PSFs have the same intensity distributions as those discussed in section 2.3, and we add background noise with a peak level of 0.16, also similar to the previous sections. Figure 19(a) shows sample velocity profiles at three different streamwise locations of bubbles with *L_p_* = 263 μm when SupBD-net is used for detecting the bubble centers. While the prescribed and measured profiles are very similar, especially at the center of the vessel, they deviate in regions with high-velocity gradients near the wall, as highlighted in the inserts. The relative error in velocity measurement is defined as ${E_u}(r) = \left| {{{\left( {{U_m}(r) - {U_{ref}}(r)} \right)} \mathord{\left/ \right. } {{U_{ref}}(r)}}} \right|$, where *U_m_* (*r*) is the measured value averaged over time and along the streamwise direction, and *U_ref_* (*r*), is the prescribed parabolic velocity profile. The *E_u_* in figure 19(a) is provided in figure 19(c), showing an increasing error with increasing velocity gradients near the wall. Also shown in figure 19(c) is a plot of $\left| {{{\left( {{{d{U_{ref}}} \mathord{\left/ \right. } {dr}} \times {E_c}} \right)} \mathord{\left/ \right. } {{U_{ref}}}}} \right|$, where *E_c_* is the error in bubble center location (see section 3.1), which gives a very similar error profile, confirming that the primary contributor to the error in velocity is the misplacement of bubble centers. Note that while the actual magnitudes of the errors are small, their relative values near the wall are large owing to the low velocity there. Still, this problem needs to be corrected, and a method to address it is provided below.

The proposed solution is illustrated in figure 18(b). Once an overlapping event is detected at *t*_3_, i.e. there is only one candidate (solid star) in the vicinity of the estimated locations for two tracks, the tracking procedure skips the detected center at *t*_3_ and predicts the likely bubble locations (hollow stars) for each track at *t*_4_ using a Kalman filter [11]. For each track, if a candidate (solid star) is found near the predicted location at *t*_4_, this candidate is assigned to this track, and a piecewise bicubic interpolated value [54] is used for the missing location at *t*_3_ (white square). In cases where no candidate is found in the vicinity of the predicted location at *t*_4_, e.g. the red track in figure 18(c), this track is terminated at *t*_2_. This approach prevents the situation illustrated in figure 18(a), reducing the likelihood of prescribing the wrong velocity in regions with overlapping PSFs. We have re-analyzed the synthetic data using the modified tracking procedure. Sample velocity profiles are presented in figure 19(b), and the corresponding *E_u_* is included in figure 19(c). As is evident, the modified procedure is considerably more effective in reproducing the parabolic velocity profile, and the region with a relative error larger than 5% is confined to the outer 6% of the radius.

**Figure 20.**
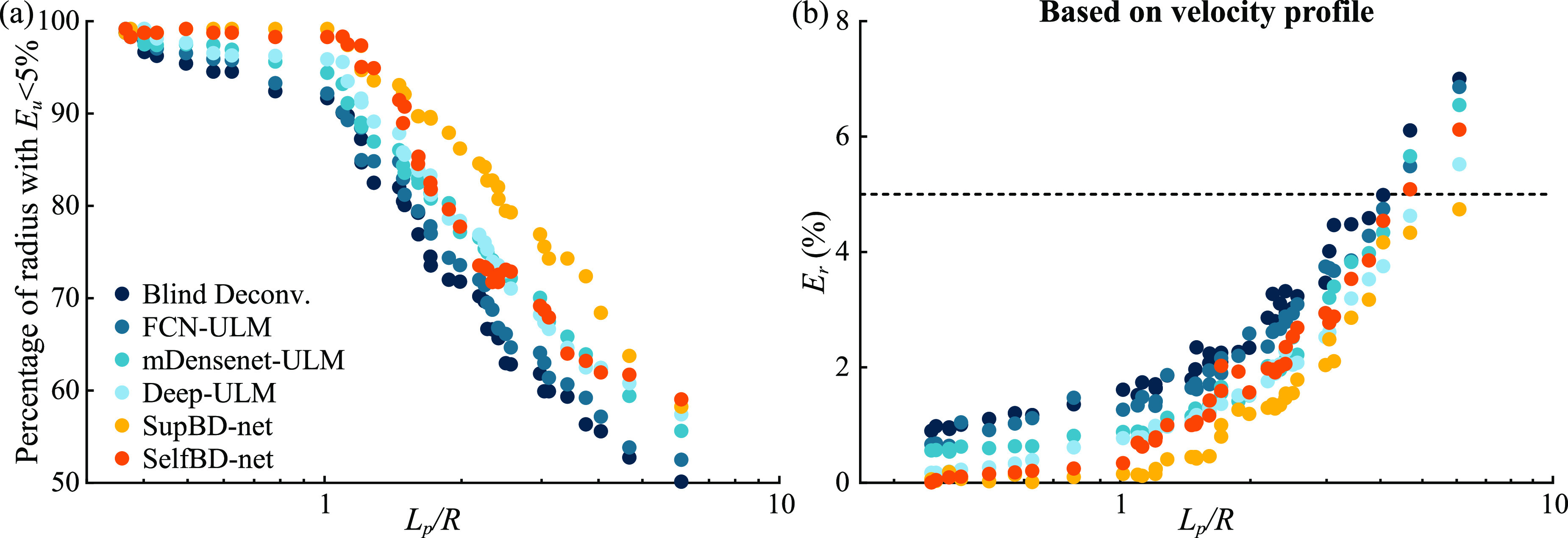
The variations with normalized PSF size *L_p_/R* of: (a) the percentage of the radius with *E_u_* < 5% and (b) the relative error in radius (*E_r_*) measured based on the velocity profile. The vertical axis is in linear scale while the horizontal axis is in log scale.

To complete the analysis, figure 20(a) shows the effect of PSF size relative to the vessel radius, namely *L_p_/R*, on the percentage of the radial locations with an *E_u_* lower than 5%. Results are provided for all the present image processing procedures and for the major axes of the PSFs aligned with or perpendicular to the axis of the pipe. The improved detection of bubble center by SupBD-net improves the measurements of velocity profiles and vessel diameter in large vessels. As expected, for all imaging procedures, the fraction of regions with *E_u_* < 5% decrease sharply with increasing *L_p_/R* once it is larger than 1, with SupBD-net having the least regions with erroneous velocity. Most notably, for the SupBD-net and SelfBD-net data, the radius fraction with *E_u_* < 5% approaches 100% for *L_p_/R* < 1. For all cases, they approach 100% when *L_p_/R* < 0.4. Figure 20(b) presents the error in the vessel radius, i.e. ${E_r} = \left| {\left( {{R_m} - R} \right)/R} \right|$, where *R_m_* is the radius estimated based on the zero crossings of parabolic fits to the velocity profiles, and *R* is the prescribed radius. For all the image processing methods, *E_r_* increases with increasing *L_p_/R*, with the SupBD-net giving the least error, and blind deconvolution, the worst one. For example, for SupBD-net the errors remain below 5% for all *L_p_/R*, whereas in the blind deconvolution data, *E_r_* exceeds 5% when *L_p_/R* > 4.

**Figure 21.**
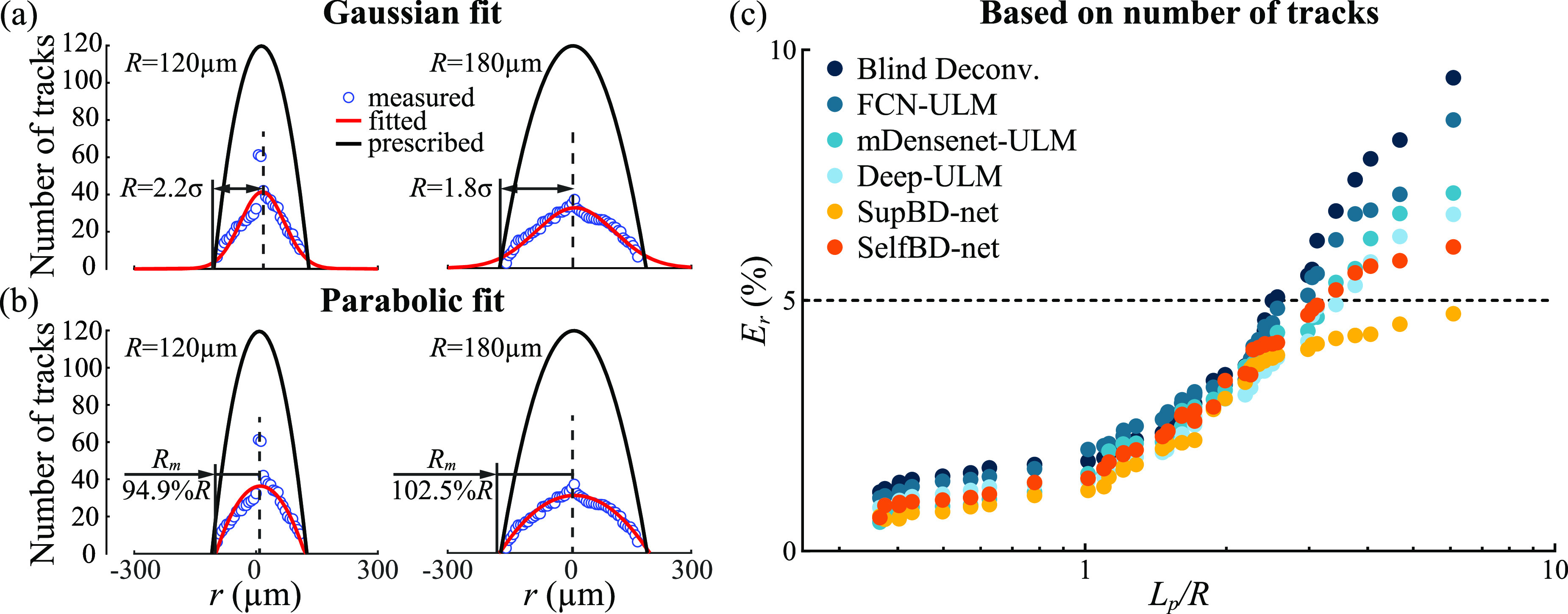
Measurement of the vessel radius based on the profiles of the number of bubble tracks. (a) Gaussian fitting (red curve) to the number of tracks (blue circles) showing inconsistent length which matches the prescribed profile (black curve). (b) The zero crossings of parabolic fitting showing better consistency with the prescribed radius. (c) The variations with *L_p_/R* of *E_r_* measured based on the number of tracks and parabolic fit. The vertical axis is in linear scale while the horizontal axis is in log scale.

Alternatively, the radius of the macro vessel could also be determined based on the profiles of the number of tracks across this vessel. As a first attempt, in figure 21(a), we fit Gaussian profiles to the measured number of tracks in vessels with radii of 120 μm and 180 μm. As is evident, the prescribed radii correspond to different multiples of the standard deviation of the Gaussian fits that differ from each other by as much as 20%, suggesting that these fits might not be the best choice for determining the vessel radius. In contrast, as demonstrated in figure 21(b), parabolic fits to the same track profiles give zero crossings that fall below 5% of the prescribed radii. Hence, parabolic zero crossings seem to be a better curve-fitting choice. However, one should keep in mind that in the present synthetic analysis, the bubbles are uniformly distributed, resulting, because of the velocity distribution, in a parabolic number of tracks. The track profile might be different in real biological systems owing to, e.g. non-parabolic velocity profiles in curved vessels or radial migration of bubbles. Using the parabolic fits, figure 21(c) shows the distribution of *E_r_* as a function of *L_p_/R* for all the present processing techniques. All the methods have similar relative errors for *L_p_/R*< 2.5, but they diverge for larger PSFs, with SupBD-net, which remains under 5% for all the present ranges, giving the best results. If a 5% error in radius is acceptable, SupBD-net-based procedures can be reliably used for measuring the velocity profiles and determining the blood vessel radius even when the PSF is significantly larger than the vessel radius.

### 3.5. Sample visualizations of the cerebral microvasculature

**Figure 22.**
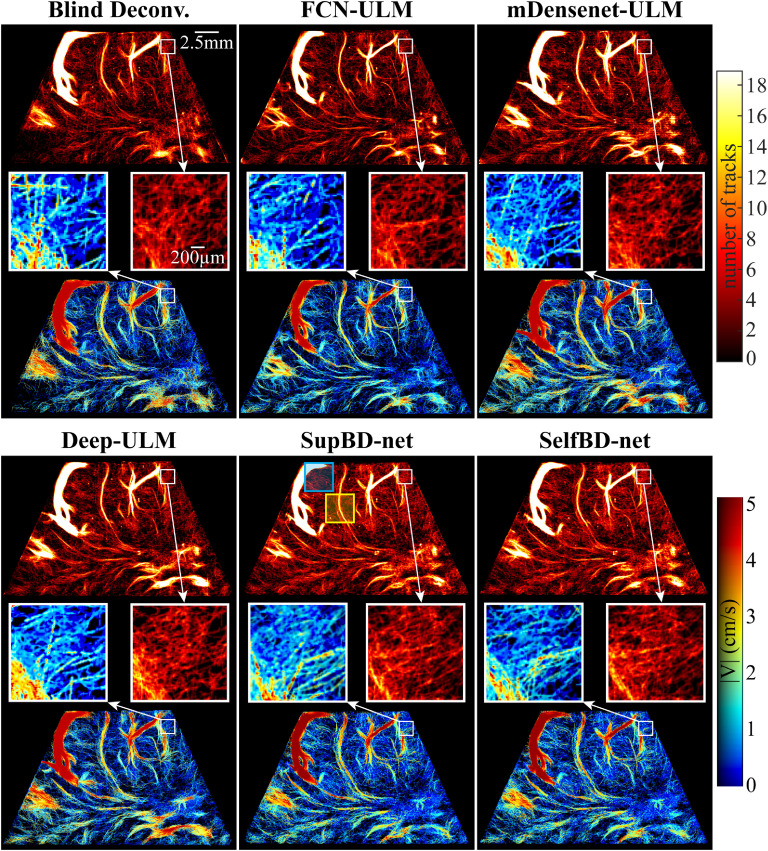
Sample heat maps and velocity distributions of the cortical vasculature in the left cerebral hemisphere of a piglet measured based on the indicated methods. For each panel: top row: heat maps of the number of bubble trajectories; bottom row: corresponding maps of average blood velocity; and middle row: heat maps and velocity distributions in the highlighted magnified subregion enclosed by the white box.

**Figure 23.**
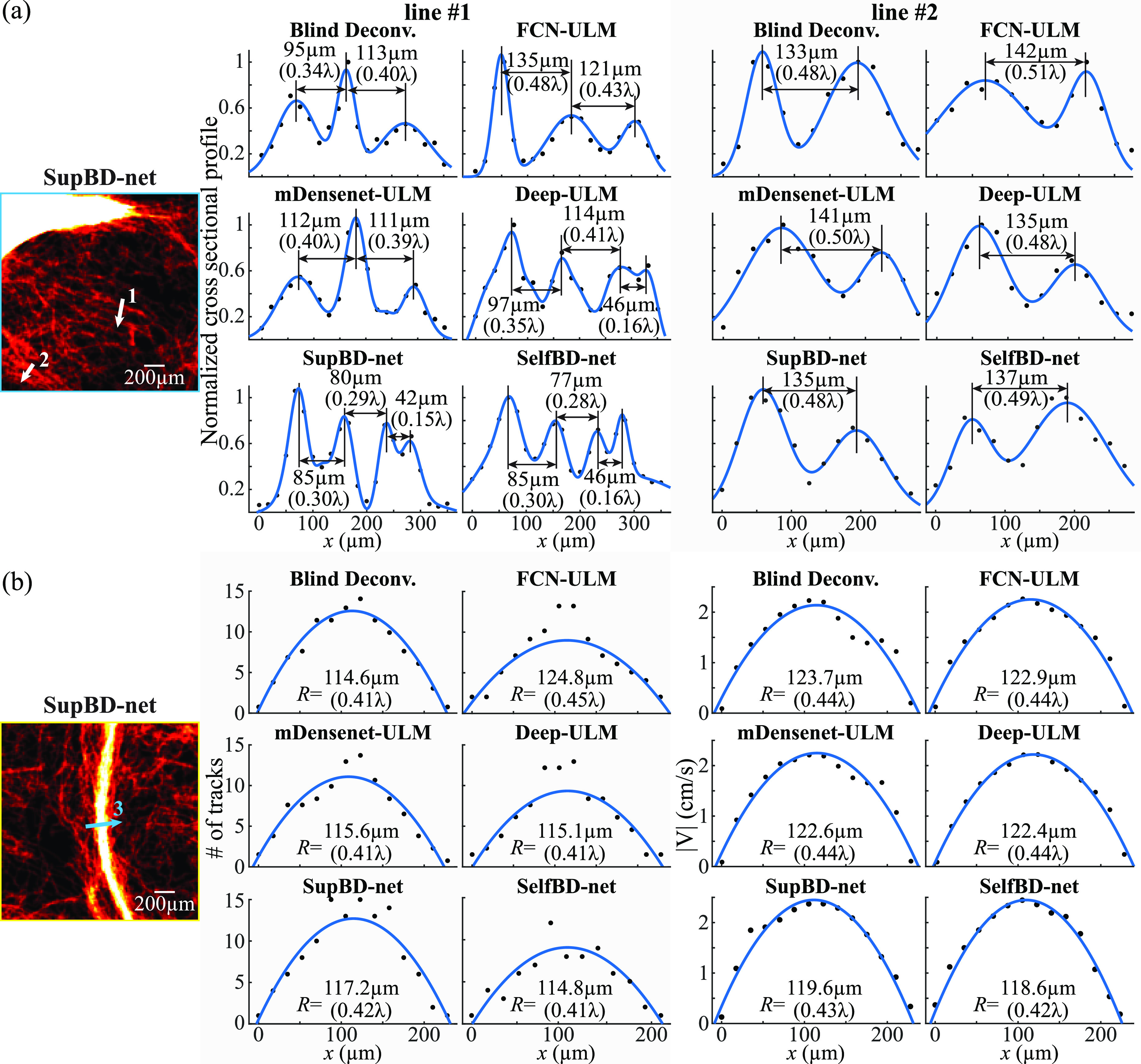
(a) A magnified view of the region in the blue box of figure 22. Two cross-sectional profiles along four closely spaced microvessels (line 1) and two moderately spaced microvessels (line 2) are provided for all methods to compare the spatial resolution of different methods. (b) A magnified view of the region in the yellow box of figure 22, where the cross-sectional profile along a moderate blood vessel (line 3) is provided for all methods comparing their abilities to estimate the radius of this vessel. The left and right panels show the radius estimation based on the parabolic fit to the number of tracks and velocity profile, respectively.

This section provides and analyzes sample maps of the cerebral cortex vasculature obtained based on 7000 CEUS images of a piglet’s brain. Heatmaps of the number of bubble trajectories highlighting cortical vessels are presented in the top row of each panel in figure 22, and the corresponding distributions of velocity magnitude are presented in the bottom row. The middle row contains magnified heatmaps and velocity distributions in the 1.5 × 1.5 mm^2^ area enclosed by the white box. For vessels with diameters exceeding 400 μm (1.4 *λ*), all the methods appear to give consistent heatmaps and velocity distributions. However, as the magnified sections demonstrate, there are noticeable differences in the location, concentration, and velocity in smaller vessels, where the Deep-ULM, SupBD-net, and SelfBD-net results are similar and appear to contain more detected microvessels than those generated using blind deconvolution and FCN-ULM. These trends are consistent with the higher resolution and reduced errors associated with SupBD-net and SelfBD-net. Sample comparisons between profiles of the number of tracks along two lines located in the blue-enclosed area (figure 22), denoted as lines 1 and 2, are presented in figure 23(a). Each plot is normalized by its maximum value, and in this region, *L_p_* is about 270 μm (0.96 *λ*), i.e. about equal to the entire length of the line sections. Based on the SupBD-net and SelfBD-net heat map profiles, line 1 cuts through four closely spaced vessels separated, based on the SupBD-net data, by 85 μm (0.30 *λ*), 80 μm (0.29 *λ*), and 42 μm (0.15 *λ*) from left to right. Deep-ULM can separate the left two vessels but with larger spacings, and it can marginally distinguish the right two ones, but with significant overlap. Blind deconvolution, FCN-ULM, and mDensenet-ULM can detect the left two vessels but not the more closely located right-most vessels. These trends are consistent with the classifications depicted in figure 17, where vessels separated for more than 0.25 *λ* can be readily distinguished by all methods. As for line 2, all the methods show two lines with spacing in the 133–142 μm (0.48–0.51 *λ*) range, also consistent with the separation limits presented in figure 17. The maximum difference in spacing among all techniques, about 9 μm, is also consistent with the error estimates for *L_p_/D_l_* ∼ 2 (figure 15), namely 6 μm for blind deconvolution and 3 μm for SupBD-net. In summary, the differences in detection abilities for microvessels are consistent with the limits in line spacing and detection errors determined based on the synthetic data.

Finally, figure 23(b) compares estimations of the radius of a moderate size blood vessel inside the yellow enclosed region (figure 22) based on parabolic fits to the heat maps shown on the left side, and the velocity distributions presented on the right side. Although the detected number of tracks varies among the different methods, the measured peak velocities are similar, with values around 2.4 cm s^−1^. In this case, *L_p_/R* ∼ 3, hence the expected velocity-based errors in radius should be 2%–4% (figure 20(b)), and those based on the heat maps should vary between 4%–6% (figure 21(c)). In general, all the heat map-based results are lower than those based on velocity, with the highest discrepancies occurring for blind deconvolution. The smallest discrepancy, less than 2%, occurs for SupBD-net. The variations among velocity-based estimates (∼2%) are (slightly) smaller than those based on the heat maps (∼4%), in accordance with their expected accuracy trends.

## 4. Conclusions

Aiming to improve the accuracy of flow measurements and vascular mapping in ULM, this study introduces and applies several machine learning methods to improve the subpixel accuracy in detecting the center of microbubble traces in CEUS images. A key to accurate localization is the determination of the 2D intensity distribution of the spatially varying PSF whose typical size is two orders of magnitude larger than the bubble diameter, and in many cases, even larger than the entire blood vessel. Adopting available deep learning building blocks, we develop supervised and self-supervised super-resolution methods named SupBD-net and SelfBD-net, respectively. SupBD-net uses local and global residual learning structures for extracting the super-resolved bubble center locations at a resolution of *λ*/14. SelfBD-net extends the generalizability of the pretrained super-resolution network by introducing a new loss function that enables reliable estimation of unknown PSFs. In this case, the analysis is only based on the raw images, without reference to the actual location of the bubble center and the PSF shape. Their performances are compared to those of the recently introduced supervised learning frameworks namely FCN-ULM, mDensenet-ULM, and Deep-ULM, as well as the more conventional blind deconvolution technique, which has been used in multiple applications of ULM.

For all cases, training and evaluation are based on synthetic CEUS images with realistic PSF shapes, background noises, and bubble concentrations. The comparison between methods includes the ability to locate a single bubble, separate closely located bubbles, distinguish between neighboring microvessels, as well as measure the velocity distribution and radius of macro vessels. For all methods, the errors in bubble center location increase with PSF size, background noise level, and bubble density. In general, SupBD-net gives the best results, followed closely by SelfBD-net. The errors yielded by the other supervised learning methods and blind deconvolution are typically 35%–82% higher. For the ideal case, where there is only one bubble without any noise, SupBD-net yields an error in the location of the bubble center of about 0.03 *λ*, and about half of the error of blind deconvolution. Even for the highest bubble concentration, PSF size, and noise level, the SupBD-net localization errors remain smaller than 0.1 *λ* (subpixel level), while those of the other methods exceed 0.12 *λ*. For closely located bubbles, SupBD-net and SelfBD-net can separate more than 95% of them if the distance between their centers exceeds 60% of the local PSF size, while other methods require separation distances of 70%–100%. These findings suggest that residual learning, the key feature in both SupBD-net and SelfBD-net, systematically gives superior detection performance. The self-supervised learning capability of SelfBD-net has been evaluated based on synthetic images with elliptical PSFs of varying aspect ratios rotated by 90 degrees. While the accuracy of SupBD-net along with the other supervised learning methods degrade at all PSF aspect ratios, and fail when they exceed 1.81 and 1.5, respectively, SelfBD-net can still detect the bubble center at an accuracy of less than 0.15 *λ*, and estimate the shape of the unknown PSF. Clearly, SelfBD-net appears to be more robust than SupBD-net in cases involving very different PSFs, making it the preferred approach when used with ultrasound systems that are different from those of the training set.

For cases involving closely located parallel microvessels, as the distance between them decreases, the cross-sectional profile of the number of tracks changes from a pair of fully separated peaks to two maxima with a ghost peak between them that increases in amplitude with decreasing distance between vessels, and to merged tracks. The ghost peaks occur when the PSFs of two bubbles partially overlap, and the image processing methods fail to separate them. Since ghost peaks might be mistakenly identified as actual microvessels, a SVM is used for introducing criteria for distinguishing between negligible and non-negligible ghost lines based on the *AF* and the *ID* of lines. The resulting heat maps define the relationship between the PSF size and the ability to distinguish between lines. As a general conclusion, the performance of SupBD-net is superior for all PSF sizes, including those PSFs that are several times larger than the line spacing. For example, SupBD-net can distinguish two vessels separated by more than 45 μm (0.16 *λ*) for PSFs as large as 500 μm. In contrast, for the same PSF, blind deconvolution requires a minimum separation spacing of 150 μm (0.54 *λ*). For all the methods, the proximity of two lines also causes errors in locating them that increase with PSF sizes. Plotting these errors vs. the PSF size normalized by the vessel spacing shows power laws that vary with the processing tools. Owing to its superior ability to separate closely located bubbles, SupBD-net has the lowest errors, especially for large PSFs, followed by SelfBD-net, Deep-ULM, mDensenet-ULM, FCN-ULM, and blind deconvolution.

Differences in the ability to separate closely located tracks also affect the measurement accuracy of velocity profiles and vessel sizes for macro vessels. We show that an erroneous shift in the location of the bubble center caused by the merging of PSFs results in significant errors in the velocity near the vessel wall. Consequently, a simple interpolation procedure is introduced, which completes the wrongly terminated tracks, significantly reducing the error in velocity. Once implemented, for a PSF comparable to or smaller than the vessel radius, SupBD-net can maintain an error in velocity of less than 5% at the wall and 0.5% at the center of the vessel. The vessel radius is estimated by fitting parabolic profiles to the velocity profile and to the number of bubble tracks. Using either approach, SupBD-net maintains an error of less than 5% even for PSFs sizes as large as five times the radius. The errors associated with the other techniques, especially blind deconvolution, are higher, increasing with PSF size. However, all methods give reliable results for PSFs smaller than the vessel radius. Consistent results in micro and macro vessels are also obtained when the analysis is based on actual CEUS data that are collected in the cortex of a neonatal pig by a clinical ultrasound scanner. It should be noted that the present analysis is based on straight vessels, and should be extended (in future studies) to non-parallel trajectories occurring e.g. in branching or complex vascular structures.

In summary, the residual learning based supervised deep learning method proposed in this work shows superior sub wavelength accuracy in detecting the location of microbubbles in ULM, resulting in improved spatial resolution of closely located microvessels, as well as accurate measurement of the velocity and radius of macro vessels. Moreover, the self-supervised method can correctly estimate the unknown PSFs and detect bubble centers, even when the supervised learning methods fail. Hence, applications of these techniques would improve the spatial resolution and accuracy of ULM analysis.

## Data Availability

The data that support the findings of this study are openly available at the following URL/DOI: 10.5281/zenodo.8256554 [55].
